# Ultra‐Low Density Covalent Organic Framework Sponges with Exceptional Compression and Functional Performance

**DOI:** 10.1002/anie.202502513

**Published:** 2025-03-27

**Authors:** Chenhui Ding, Yingying Du, Tamara Fischer, Jürgen Senker, Seema Agarwal

**Affiliations:** ^1^ Macromolecular Chemistry and Bavarian Polymer Institute University of Bayreuth Universitätsstrasse 30 Bayreuth 95440 Germany; ^2^ Department of Chemistry Inorganic Chemistry III, and Northern Bavarian NMR Centre University of Bayreuth Universitätsstrasse 30 Bayreuth 95440 Germany

**Keywords:** Absorption, Catalytic, Compressible, Covalent organic frameworks, Sponge, Ultralow density

## Abstract

The emergence of covalent organic frameworks (COFs) macroscopic objects with hierarchical porous structures addresses the limitations of traditional COF powders, which are challenging to process, thus bringing them closer to practical applications. However, the brittleness of the parent COF powder results in poor mechanical stability of these COF macroscopic objects, presenting a significant challenge that must be overcome for their continued development. In this work, we successfully obtained a continuous, hierarchically porous, and interconnected open‐cell COF structure made up of hollow sponge walls of thickness 100–250 nm through a template‐assisted framework process. This unique structure endows the COF sponge with a high surface area (1655 m^2^ g^−1^), ultralow density (2.2 mg cm^−3^), and exceptional mechanical stability. Even after 300 000 compressions at a 50% compression rate, its stress and height decreased by only 7.9% and 7.1%, respectively. These properties grant the COF sponge excellent solvent absorption capacity, catalytic performance, and reusability. Therefore, this work broadens the development pathway for COF macroscopic objects and is expected to further unlock the potential of COFs in practical applications.

## Graphical abstract

The construction method for the hierarchical porous covalent organic framework (COF) sponges with hollow cell walls through a template‐assisted framework solvothermal process is shown. The COF sponges exhibit ultralow density (2.2 mg cm^−3^), high surface area (1655 m^2^ g^−1^), and unprecedented compression performance (demonstrated by only a 7.9% decrease in height and a 7.1% decrease in stress after 300 000 compressions at 50% strain). Excellent performance and reusability in organic solvent absorption and organic catalysis are demonstrated.

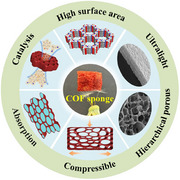

## Introduction

Covalent organic frameworks (COFs) are porous crystalline cross‐linked materials.^[^ ^1^, ^2^, ^3^, ^4^ ^]^ They have customizable porous structures and functions, exceptional stability, and large surface areas, making them valuable in various fields such as separation,^[^ ^5^, ^6^, ^7^, ^8^ ^]^ catalysis,^[^ ^9^, ^10^, ^11^ ^]^ energy storage,^[^ ^12^, ^13^, ^14^, ^15^ ^]^ environmental remediation,^[^ ^16^, ^17^, ^18^ ^]^ and biomedical engineering.^[^ ^19^, ^20^, ^21^ ^]^ However, COFs typically exist as microcrystalline powders, which are insoluble, nonmelting, and difficult to process into required forms. This presents challenges in the separation and recycling of COF powders during use. Additionally, the agglomeration of COF powders impedes substance transport and limits their accessibility.^[^ ^22^, ^23^, ^24^, ^25^ ^]^ To address these issues, several methods have been developed to process COFs into macroscopic objects, including 2D films or membranes^[^ ^26^, ^27^, ^28^, ^29^ ^]^ and 3D macroscopic objects (aerogels, sponges, and foams).^[^ ^30^, ^31^, ^32^ ^]^ Among these, 3D macroscopic objects are particularly advantageous as they not only retain the intrinsic properties of COFs but also introduce hierarchical porous structures. These structures facilitate substance transport and exposed active sites, effectively broadening the potential applications of COFs.^[^ ^33^, ^34^, ^35^ ^]^

Generally, COF macroscopic objects can be categorized into COF‐based materials and pure COF materials. COF‐based macroscopic objects are typically formed by encapsulating COF particle within a polymer network (such as chitosan and poly(vinyl alcohol))^[^ ^36^, ^37^, ^38^ ^]^ or by growing COFs on the surface of a carrier (like graphene aerogel, short fiber sponge, and melamine foam).^[^ ^39^, ^40^, ^41^ ^]^ The introduction of a carrier imparts the COF‐based macroscopic objects with a hierarchical porous structure and excellent mechanical stability. The reliance on a carrier to form the macroscopic objects limits the potential for further development and application of COF macroscopic objects.

In contrast, pure COF macroscopic objects, constructed entirely from COFs, can perfectly circumvent these issues. These materials have seen rapid development in recent years, with various strategies emerging, including 3D printing,^[^ ^42^, ^43^ ^]^ in‐situ gas phase foaming,^[^ ^44^ ^]^ sol–gel methods,^[^ ^45^, ^46^ ^]^ and nanoparticle/microparticle template‐assisted techniques.^[^ ^47^, ^48^ ^]^ However, pure COF macroscopic objects reported thus far are usually composed of COF particles, which suffer from weak interactions between the particles and a high number of defects. When subjected to external forces, these structures often collapse, losing both their initial macroscopic shape and internal hierarchical porous structure.^[^ ^49^, ^50^, ^51^ ^]^ Therefore, developing pure COF macroscopic objects with both mechanical stability and well‐defined hierarchical porous structures remains a significant challenge in the field, which we have solved in the present work using a template‐assisted framework (TFA) solvothermal process.^[^ ^52^ ^]^

Specifically, a hierarchical porous polymer template sponge was first prepared via freeze‐drying. A large number of COF nanoparticles were then grown in situ on its surface, closely stacking to form a continuous COF film with a thickness of 100–250 nm. After removing the polymer template, a continuous, hierarchically porous, and hollow pure COF sponge was obtained. This unique structure endows the COF sponge with several remarkable properties, ultralow density (2.2 mg cm^−3^, the lowest density reported for COF macroscopic objects to date), high surface area (1655 m^2^ g^−1^), and excellent mechanical stability (demonstrated by only a 7.9% decrease in height and a 7.1% decrease in stress after 300 000 compressions at 50% strain). This further enhances the COF sponge's performance in various applications: 1) It possesses the highest solvent absorption capacity among known COF materials, allowing for quick and efficient solvent discharge through simple squeezing, enabling multiple and efficient reuse; 2) it accelerates material transfer, exposing more catalytic sites, and demonstrates catalytic activity far surpassing that of COF powder as shown exemplarily in the Knoevenagel reaction. Remarkably, even after 20 cycles of use, the COF sponge maintains its solvent absorption capacity, catalytic performance, and overall structure, demonstrating excellent durability and reusability. These characteristics position the COF sponge as a future material for various practical applications, fully unleashing its potential in different scenarios.

## Results and Discussion

**Figure 1:**
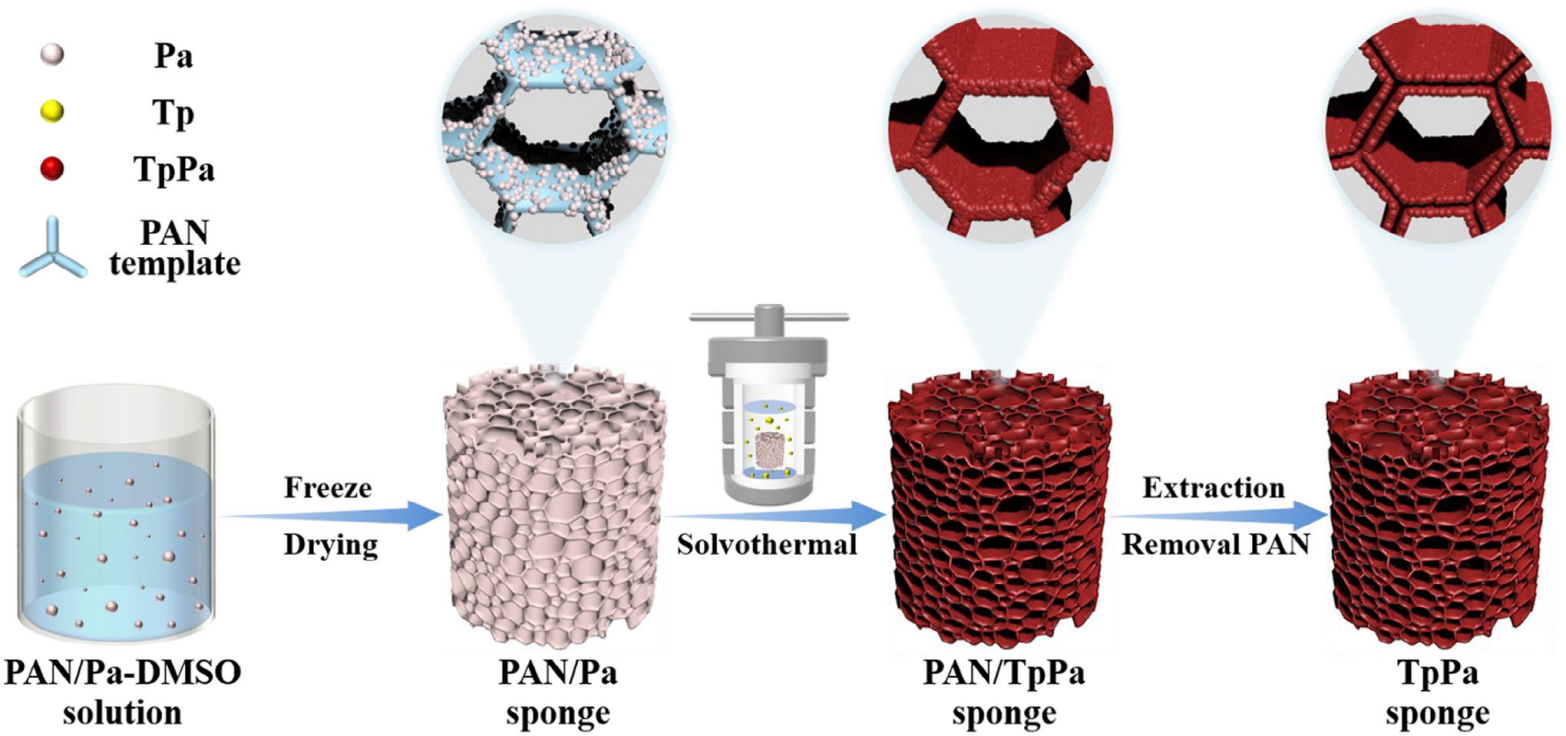
Schematic of the preparation strategy used in this work for TpPa sponges.

The most commonly used imine‐linked COF, TpPa (Figure S1), was selected as the research model. Polyacrylonitrile (PAN) was used as the template polymer, and a hierarchical porous and hollow TpPa sponge was prepared using the TFA solvothermal process, as shown in Figure 1.

**Figure 2:**
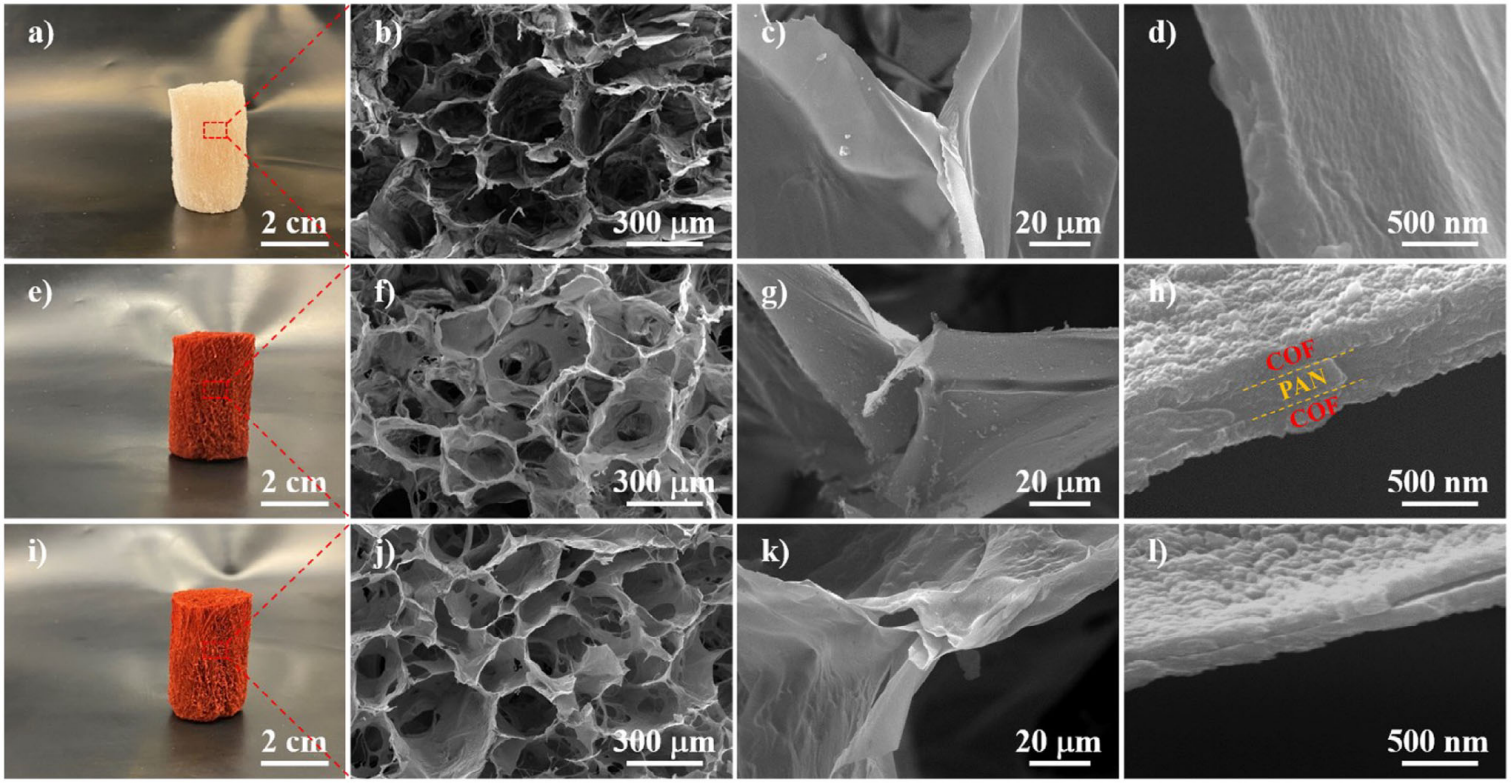
Photographs and SEM images of PAN/Pa sponge a)–d), PAN/TpPa sponge e)–h), and TpPa sponge i)–l).

Initially, p‐phenylenediamine (Pa) was dissolved in a specific concentration in a uniform solution of PAN in dimethyl sulfoxide (DMSO). This solution was then freeze‐dried, resulting in a light‐pink PAN/Pa open‐cell sponge (Figures 2a–d). The external macroscopic morphology of the sponge was determined by the mold used during the freezing process, yielding a cylindrical shape in this study. Internally, the sponge exhibited a continuous hierarchical porous structure, with the PAN loaded with Pa, making the sponge cell walls provide an ideal platform for the in‐situ growth of TpPa.

After this, the sponge was soaked in a dichloromethane solution containing 1,3,5‐triformylphloroglucinol (Tp) and subjected to solvothermal reaction at 120 °C for 1day, with acetic acid serving as a catalyst. During this process, Pa reacted with Tp, resulting in the in‐situ growth of numerous TpPa nanoparticles on the PAN skeleton. These nanoparticles were tightly packed together through a symbiotic process, forming defect‐free, continuous TpPa film that completely enveloped the PAN sponge skeleton. Simultaneously, the sponge transformed into a red, hierarchically porous PAN/TpPa sponge (Figure 2e–h).

Finally, the PAN template was removed through solvent (dimethylformamide) extraction, yielding a pure TpPa sponge. The resulting TpPa sponge retained its external macroscopic morphology and color even after the PAN template was eliminated (Figure 2i). Internally, the continuous TpPa cell walls and hierarchical porous structure were preserved, forming a TpPa sponge with hollow walls (Figures 2j–l). For comparison, a red spherical TpPa powder (Figure S2), assembled from nanoparticles, was synthesized using the same solvothermal method.

Moreover, by adjusting the concentration of PAN and Pa in the DMSO solution during the preparation process (first step), different TpPa sponges could be fabricated, as summarized in Table S1. The concentration of PAN in DMSO was fixed at 10 mg mL^−1^, while the mass ratio of PAN to Pa was varied to 10:3, 10:5, and 10:10, respectively. The resulting TpPa sponges were named TpPa sponge‐3, TpPa sponge‐5, and TpPa sponge‐10. Because of the consistent concentration of PAN in the DMSO solution, the resulting TpPa sponges exhibited similar hierarchical porous structures, with an average pore size of approximately 210 µm. With the increase in Pa content, an increase in the density (from 2.2 to 6.7 mg cm^−3^) of the TpPa sponge and the thickness of its hollow skeleton TpPa cell wall (from 103 to 214 nm) was observed (Figure S3a–i). Furthermore, by increasing the concentration of PAN in DMSO to 20 mg mL^−1^ and adding the same mass of PAN as Pa, a TpPa sponge referred to as TpPa sponge‐20 was obtained. As shown in Figure S3j–l, TpPa sponge‐20 exhibited a similar hierarchical porous and hollow cell‐wall structure as other TpPa sponges but with a smaller average pore size of about 124 µm, a thicker hollow cell‐wall skeleton measuring 257 nm, and a higher density of 12.3 mg cm^−3^. Thus, by controlling the parameters during the preparation process, it is possible to obtain hierarchical porous TpPa sponges with hollow cell walls of varying densities. Notably, TpPa sponge‐3 stands out as the lowest density mechanically stable COF macroscopic object reported to date (Table S2), which is so light that it (5.8 mg) can be fully supported by just three dandelion hair (Figure S4).

**Figure 3:**
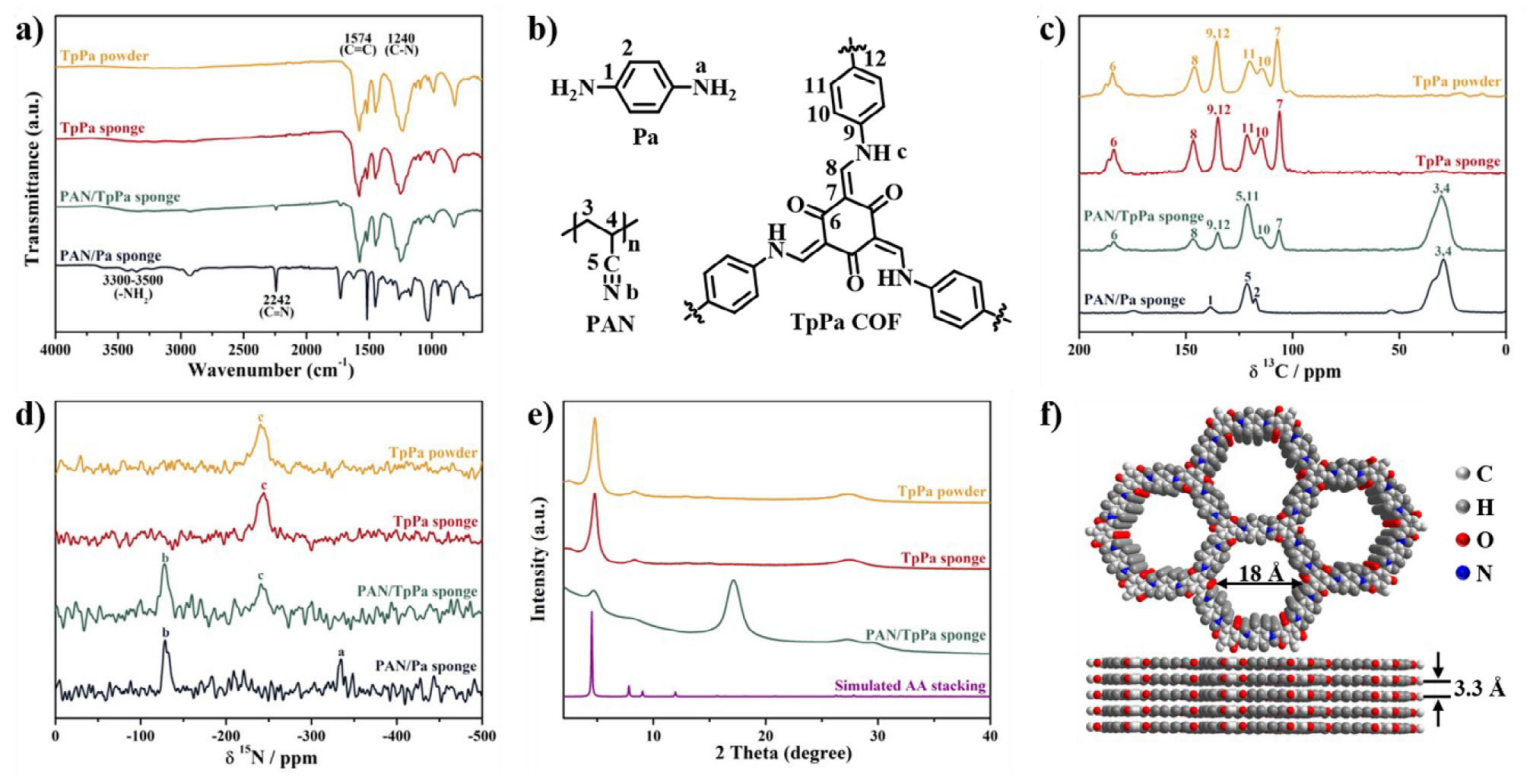
FT‐IR spectra of PAN/Pa sponge, PAN/TpPa sponge, TpPa sponge, and TpPa powder a). Chemical structural formulas of Pa, PAN, and TpPa b). ^13^C and ^15^N CP MAS NMR spectra of PAN/Pa sponge, PAN/TpPa sponge, TpPa sponge, and TpPa powder c), d). XRD patterns of PAN/TpPa sponge, TpPa sponge, TpPa powder, and the simulated AA eclipsed stacking model e). Space‐filled model of TpPa in AA stacking mode f).

Fourier Transform Infrared (FT‐IR) spectra, ^13^C and ^15 ^N cross‐polarized magic angle spinning (CP‐MAS) solid‐state NMR spectra, and X‐ray diffraction (XRD) patterns were utilized to investigate changes in the chemical and crystalline structures of TpPa sponges. The FT‐IR spectra (Figures 3a, S5a) revealed that upon completing the solvothermal reaction, the PAN/Pa sponge transformed into a PAN/TpPa sponge. This conversion was evidenced by the disappearance of the N─H (3300–3400 cm^−1^) stretching peak of Pa and the emergence of stretching peaks at 1574 cm^−1^ (C═C) and 1236 cm^−1^ (C─N), consistent with TpPa powder confirming the successful synthesis of TpPa COF.^[^ ^53^, ^54^ ^]^ After solvent extraction, the stretching peak of the PAN at 2242 cm^−1^ (C≡N) was entirely absent, while the chemical structure of TpPa was completely retained, indicating that the TpPa sponge was successfully formed. Additional evidence came from ^13^C and ^15 ^N CP‐MAS solid‐state NMR spectra (Figures 3c,d). The chemical structures with numbered carbon atoms and their corresponding NMR spectra are provided in Figure 3b. The PAN/Pa sponge showed carbon of the aliphatic main chain of PAN at ∼30 ppm and a nitrile nitrogen peak at ∼127 ppm, as well as Pa, exhibiting aromatic CH groups at ∼139 ppm and an amino nitrogen peak at ∼328 ppm. In contrast, the PAN/TpPa sponge displayed the disappearance of Pa‐specific peaks, with the appearance of TpPa‐specific imine carbon (∼147 ppm), carbonyl carbon (∼184 ppm), and keto‐enamine nitrogen (∼241 ppm) peaks. After the removal of the PAN template from PAN/TpPa sponge, all PAN‐associated peaks vanished, while TpPa peaks were retained, aligning perfectly with the spectra of TpPa powder.^[^ ^52^ ^]^

The XRD patterns provided further confirmation of the crystallinity and successful formation of the TpPa sponge (Figures 3e, S5b). The characteristic PAN reflex at 2*θ* = 17.2° was absent in the TpPa sponge, confirming the complete removal of PAN. The XRD patterns of TpPa powder, PAN/TpPa sponge, and TpPa sponge displayed clear reflections at 2*θ* = 4.8° and 27°, consistent with the AA stacking model simulation of TpPa, demonstrating high crystallinity. Based on Bragg's law, the reflections from the (100) and (001) planes indicated a pore size and interlayer spacing of approximately 1.8 and 0.33 nm, respectively (Figure 3f), in agreement with previously reported values in the literature.^[^ ^55^, ^56^ ^]^

The surface area, pore size distribution, and cumulative pore volume of the TpPa sponge and TpPa powder were analyzed using N_2_ physical adsorption isotherms at 77 K, as shown in Figure S6. All samples exhibited typical type‐I reversible isotherms, characterized by a sharp increase in adsorption at low relative pressures, indicating a highly microporous structure.^[^ ^57^ ^]^ The BET (Brunauer–Emmett–Teller) surface area of TpPa powder was measured to be 561 m^2^ g^−1^, with a pore size distribution centered around 1.8 nm, consistent with XRD patterns results, and a maximum cumulative pore volume of 0.28 cm^3^ g^−1^, which aligns well with previously reported values.^[^ ^55^, ^58^ ^]^ In comparison, the TpPa sponge showed a similar pore size distribution centered around 1.8 nm, but exhibited a significantly larger surface area (1359–1655 m^2^ g^−1^) and cumulative pore volume (0.53–0.67 cm^3^ g^−1^) as summarized in Table S3. Moreover, the surface area of the TpPa sponges steadily increased as the Pa content decreased. This increase is likely due to the structural differences between the TpPa sponge and TpPa powder. Although both materials are formed through the aggregation of nanoparticles, TpPa sponges have a hierarchically porous and hollow cell wall structure in which the thickness of the hollow TpPa cell wall is much thinner than that of TpPa powder. This reduced thickness exposes more accessible pore space in the sponge, leading to a higher surface area and cumulative pore volume. Specifically, the TpPa sponge‐3 with the thinnest hollow cell wall has a surface area of 1655 m^2^ g^−1^ and a cumulative pore volume of 0.67 cm^3^ g^−1^, far exceeding that of TpPa powder. As the thickness of the hollow sponge skeleton wall increased, the accessible pore space decreased slightly, resulting in a modest reduction in both surface area and cumulative pore volume for the thicker cell wall TpPa sponges.

**Figure 4:**
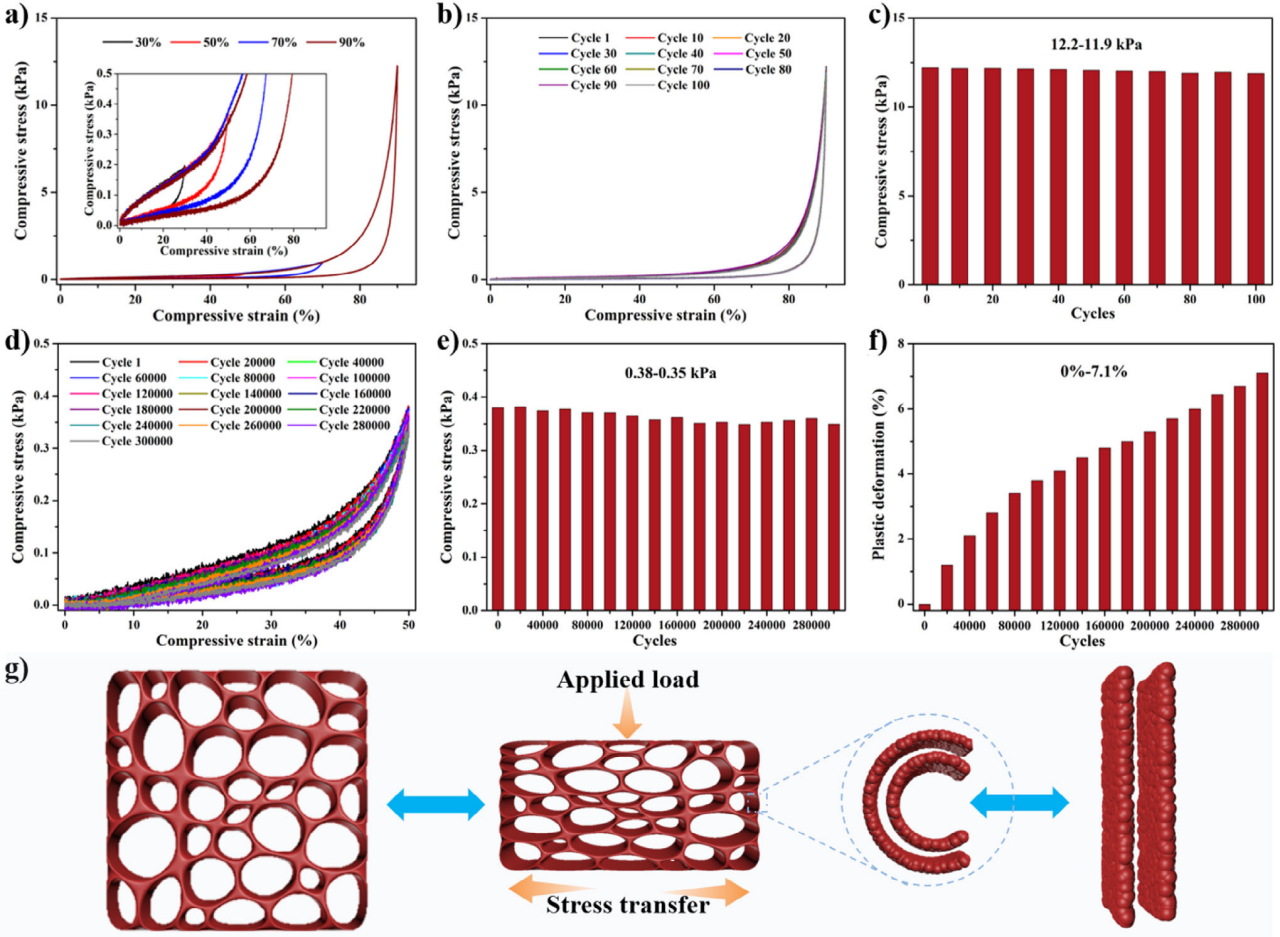
The compression stress–strain curves of TpPa sponge‐3 at different maximum strains a). The inset shows a magnification of the compression stress–strain curves. The compressive stress–strain curves b) of TpPa sponge‐3 at 90% strain for 100 cycles and cycle‐dependent compressive stress c). The compressive stress–strain curves d) of TpPa sponge‐3 at 90% strain for 300 000 cycles, cycle‐dependent compressive stress e), and cycle‐dependent height reducion rate f). Schematic diagram of the changes in TpPa sponge during compression deformation g).

The mechanical properties of the TpPa sponge were evaluated through a series of compression tests. As shown in Figure 4a, the stress–strain (σ–ε) curves of the TpPa sponge‐3 at different strain levels (30%, 50%, 70%, and 90%) show that the sponge can recover to its original shape after releasing the compression and three characteristic regions similar to elastomeric foams can be observed:^[^ ^59^, ^60^ ^]^ 1) the linear elastic region (ε < 10%), which is determined by the elastic bending of the cell wall, and the stress increases linearly with strain, but the density of TpPa sponge‐3 is too low, resulting in no obvious change in this region;^[^ ^45^ ^]^ 2) the relatively flat plateau region (10% < ε < 80%), which is determined by the elastic buckling of the cell wall, and the stress increases slowly with strain; and 3) the densification region (80%< ε < 90%), where the cell structure collapses further and the stress increases sharply with strain, indicating that the TpPa sponge‐3 is resistant to further compression. At a maximum strain of 90%, the compressive stress reached 12.2 kPa, indicating that the TpPa sponge‐3 can withstand over 35 000 times its own weight without collapsing. After 100 compression cycles at 90% strain, the stress and height of TpPa sponge‐3 remained almost unchanged (Figure 4b,c). Even when fully compressed, the TpPa sponge‐3 exhibited excellent compressive properties, returning to its original height (Video S1). To further evaluate the compression fatigue resistance, TpPa sponge‐3 was subjected to 300 000 compression cycles at 50% strain. The results showed stress decay of 7.9%, and a height reduction (plastic deformation) of only 7.1% (Figure 4d–f). Impressively, its external macrostructure and internal hierarchical porous structure remained intact after the test (Figure S7). Additionally, all TpPa sponges tested under the same conditions exhibited excellent deformation recovery (Figure S8 and Table S4). This outstanding deformation recovery and mechanical stability sets TpPa sponges apart from traditional COF macroscopic objects, which tend to exhibit brittleness,^[^ ^61^, ^62^, ^63^ ^]^ and surpass most reported 3D macroscopic objects (Figure S9 and Table S5). The superior mechanical properties of the TpPa sponge are primarily attributed to its unique structural features: 1) The efficient continuity of the internal TpPa skeleton, which prevents stress concentration and ensures even absorption and release of stress throughout the material; 2) the hierarchical porous and hollow skeleton wall structure provides ample space to accommodate high levels of deformation while effectively absorbing and releasing stress; and 3) the defect‐free continuous ultrathin TpPa cell wall (100–250 nm), which can endure significant bending while rapidly recovering its original shape (Figures 4g, S10 and Video S2). These factors combine to create a COF sponge with excellent deformation recovery properties.

**Figure 5:**
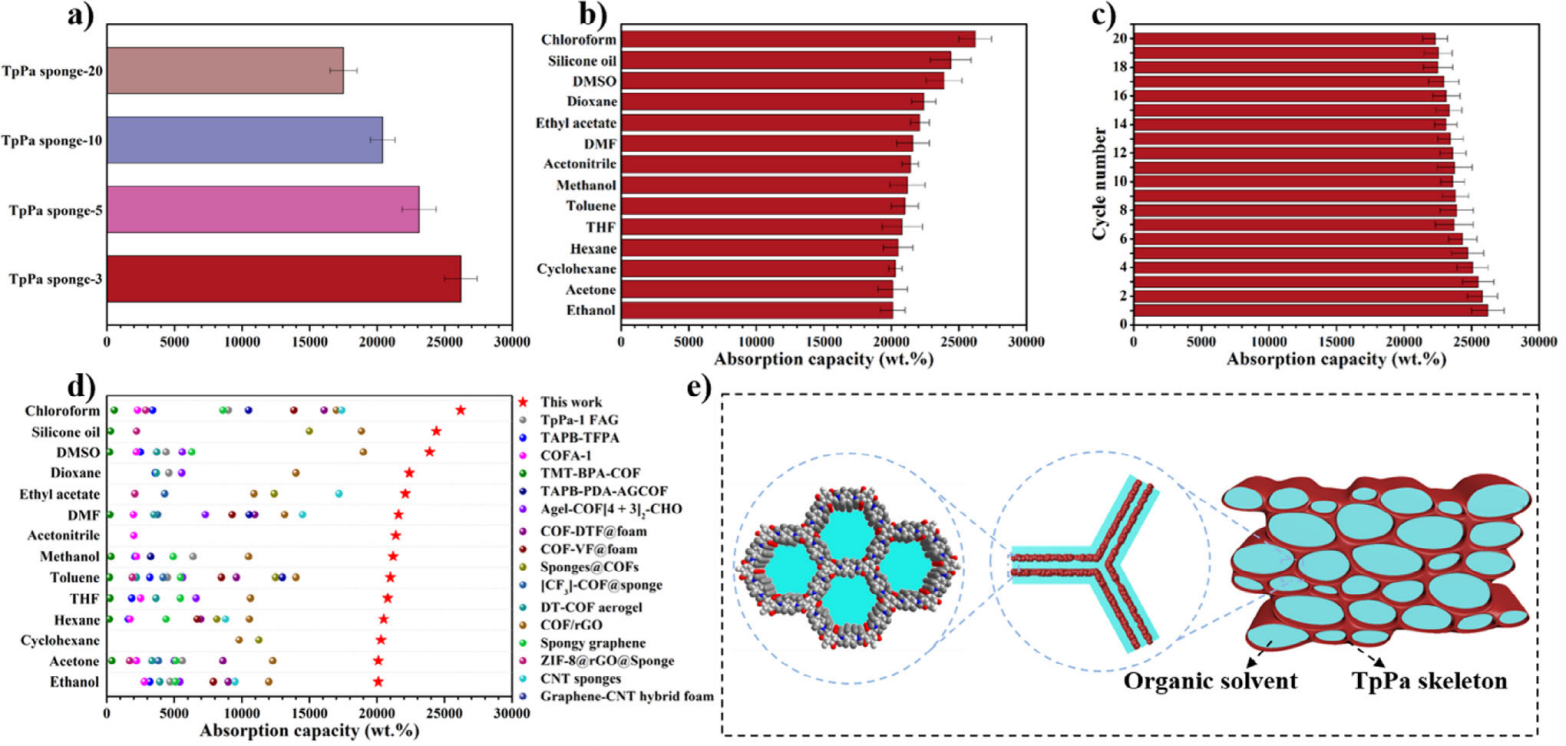
The absorption capacity of TpPa sponges for chloroform a). Absorption capacity of TpPa sponge‐3 for different organic solvents b). Performance of the TpPa sponge‐3 over 20 cycles of absorption of chloroform c). Comparison of absorption performance of TpPa sponge‐3 with other absorbents d). And a comprehensive comparison is provided in Table S6. Schematic diagram of the high‐performance absorption of organic solvents by TpPa sponges e).

TpPa sponges have hierarchical porous and hollow structures, along with their high surface area and excellent deformation recovery properties, which should make them suitable for many different applications in the future. Exemplarily we tested their performance in the absorption of organic solvents. In the case of chloroform, TpPa sponges demonstrate an exceptionally high absorption capacity, particularly TpPa sponge‐3, which can absorb 262 times its own weight (Figure 5a). Given this impressive performance, TpPa sponge‐3 was selected as the representative material to test its absorption capabilities across a range of organic solvents. The results revealed that TpPa sponge‐3 exhibited an astonishing absorption capacity for organic solvents, ranging from 201 to 262 times its own weight (Figure 5b). This absorption capacity surpasses that of previously reported COF‐based absorbents and most other absorbents (Figure 5d and Table S6). Three key factors are hypothesized to contribute to the sponge's unprecedented solvent absorption capacity: 1) The high surface area and cumulative pore volume, enable the sponge to expose more absorption sites, enhancing its overall capacity for solvent absorption; 2) the hollow TpPa cell wall structure promotes the absorption of solvents through capillary action and retains them within the sponge skeleton; and 3) the low mechanical strength causes the internal hierarchical porous structure to shrink after absorbing the solvent, and further locks the solvent between the sponge skeletons through capillary action (Figure 5e). Additionally, a significant amount of the absorbed solvent (up to 70%) can be easily expelled by simple squeezing, indicating that capillary action plays a dominant role in solvent retention. And thanks to the excellent deformation recovery ability of the TpPa sponge, it can be reimmersed in the solvent to restore its original shape, allowing for convenient and efficient repeated use without compromising its structural integrity (Video S3). Specifically, the TpPa sponge‐3 demonstrated no reduction in solvent absorption capacity across 20 cycles of use (Figure 5c). After the cycles, the external macrostructure and internal hierarchical porous structure remained fully intact, underscoring its excellent reusability and structural stability (Figure S11). This makes the TpPa sponge a promising highly durable and effective material for repeated solvent absorption applications.

**Figure 6:**
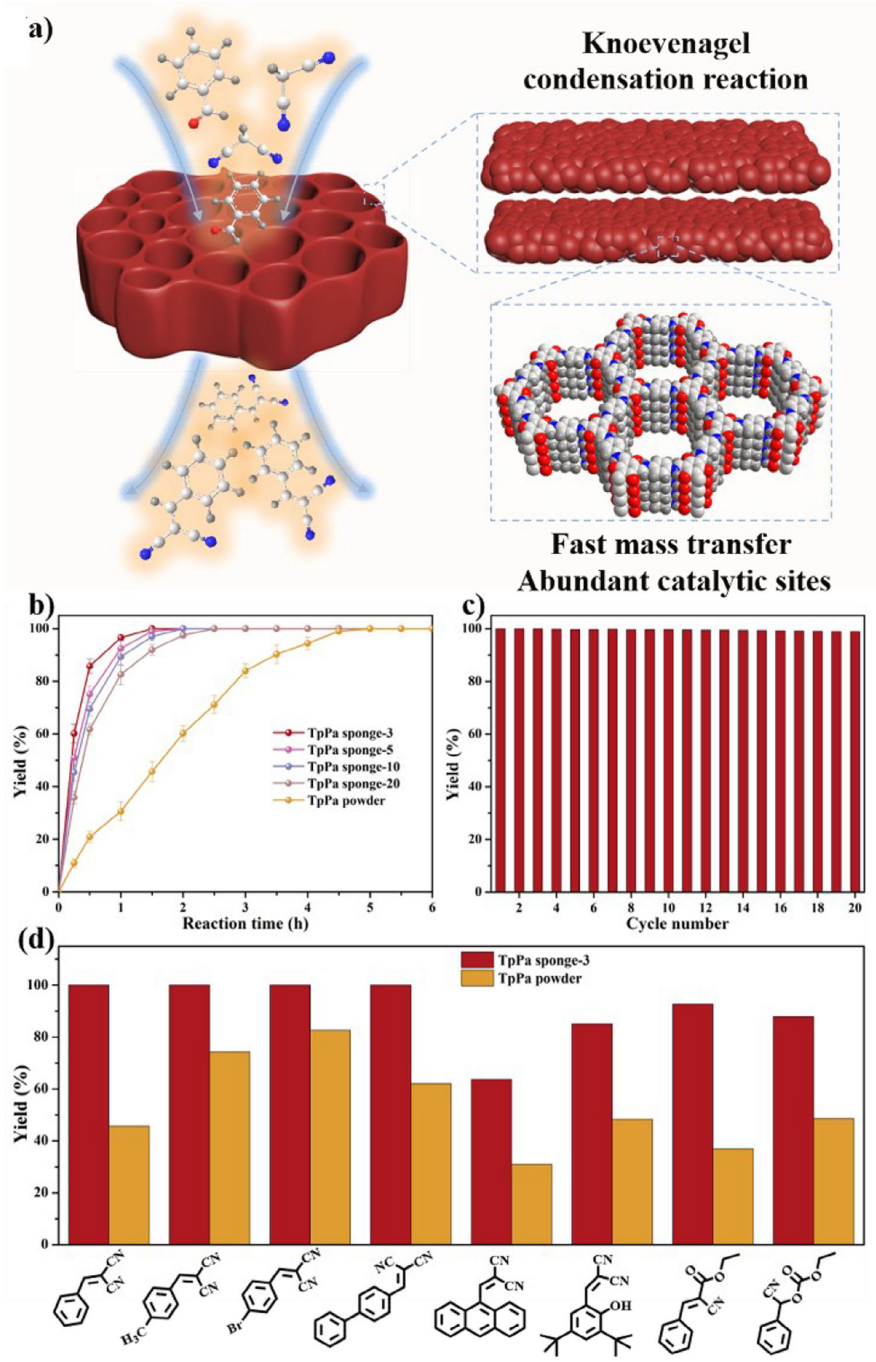
Schematic diagram of TpPa sponge catalyzed Knoevenagel condensation reaction a). Comparison of catalytic performance of different TpPa sponges and TpPa powders b). Reusability tests of TpPa sponge‐3 c). Comparison of the catalytic performance of TpPa sponge‐3 and TpPa powder in various Knoevenagel condensation reactions d). And the X axis is the corresponding products.

COF powders have been widely utilized in catalysis, but challenges such as agglomeration and difficult separation limit their broader application.^[^ ^64^, ^65^, ^66^ ^]^ In contrast, the mechanically stable TpPa sponges, with their hierarchical porous and hollow structure, ultrathin skeleton, and high surface area, offer significant advantages. These properties are expected to enhance exposure to catalytic active sites and improve mass transfer efficiency (Figure 6a). To explore this, the catalytic performance of TpPa sponges was evaluated using the Knoevenagel condensation reaction between benzaldehyde and malononitrile as a model, and their performance was compared with TpPa powders. As anticipated, all TpPa sponges demonstrated superior catalytic performance compared to TpPa powder (Figure 6b). The TpPa sponge‐3, which has the highest surface area (1655 m^2^ g^−1^) and the thinnest skeleton wall (103 nm), achieved complete conversion of benzaldehyde within 1.5 h, significantly faster than the 5 h required by TpPa powder. TpPa sponges have excellent mechanical stability and are easy to separate from the reaction solution. The reusability of the TpPa sponge‐3 was further explored, and its catalytic performance remained essentially unchanged during 20 further uses (Figure 6c). The external macroscopic morphology, internal hierarchical porous structure, chemical integrity, and crystallinity of the TpPa sponge‐3 were perfectly preserved (Figure S12), showing excellent stability. In addition, the catalytic performance of the TpPa sponge‐3 was studied in various other Knoevenagel condensation reactions, and its performance was consistently better than that of TpPa powder for all tested substrates under the same reaction conditions (Figures 6d, S13–S20, and Table S7). This highlights the superior reusability, efficiency, and stability of TpPa sponges as catalysts in heterogeneous reactions.

In addition, we successfully synthesized three other imine‐linked COF (Figure S21) sponges (TpPa‐(CH_3_)_2_ sponge, TpBD sponge, and TpBpy sponge) using the same method. FT‐IR spectra and XRD patterns confirmed that these COF sponges were obtained with good crystallinity (Figure S22). Moreover, like TpPa sponges, they also possess hierarchical porous and hollow structures, with skeleton wall thicknesses of about 200 nm (Figure S23). This demonstrates the versatility of the preparation method, suggesting its potential for broader application to a variety of COFs. This approach provides a promising pathway for the fabrication and utilization of COF macroscopic objects in various fields.

## Conclusion

This study successfully overcomes the inherent brittleness and processing challenges of COFs by developing self‐standing 3D pure crystalline COF sponges with hollow skeleton walls and varied densities and surface area through a template‐assisted framework solvothermal process. The hierarchical porous structure and hollow skeleton walls provided TpPa sponges remarkable compressive stability and demonstrated superior performance in organic solvent absorption and catalysis. The sponges maintain their structural integrity and functional efficiency across extensive reuse cycles, highlighting their potential for practical applications. Furthermore, the adaptability of the synthetic process allows for the fabrication of a diverse range of COF sponges, paving the way for future advancements in COF‐based macroscopic materials. This work establishes a robust platform for developing high‐performance COF macroscopic objects, expanding their applicability in industrial and environmental contexts. These sponges can be further modified with appropriate functional groups, tailored porosity, and a hierarchical porous structure, enabling broader applications in areas such as wastewater treatment, toxic metal ion adsorption, sensors, and catalysis.

## Conflict of Interests

The authors declare no conflict of interest.

## Supporting information



Supporting Information

Supplemental Video 1

Supplemental Video 2

Supplemental Video 3

## Data Availability

The data that support the findings of this study are available in the supplementary material of this article.

## References

[1] A. P. Côté , A. I. Benin , N. W. Ockwig , M. O'keeffe , A. J. Matzger , O. M. Yaghi , Science 2005, 310, 1166–1170. 16293756 10.1126/science.1120411

[2] X. Feng , X. Ding , D. Jiang , Chem. Soc. Rev. 2012, 41, 6010–6022. 22821129 10.1039/c2cs35157a

[3] F. Haase , B. V. Lotsch , Chem. Soc. Rev. 2020, 49, 8469. 33155009 10.1039/d0cs01027h

[4] C. S. Diercks , O. M. Yaghi , Science 2017, 355, eaal1585. 28254887 10.1126/science.aal1585

[5] S. Yuan , X. Li , J. Zhu , G. Zhang , P. v an Puyvelde , B. v an der Bruggen , Chem. Soc. Rev. 2019, 48, 2665. 31025660 10.1039/c8cs00919h

[6] Y. Ying , S. Peh , H. Yang , Z. Yang , D. Zhao , Adv. Mater. 2022, 34, 2104946. 10.1002/adma.202104946 34535914

[7] A. Knebel , J. Caro , Nat. Nanotechnol. 2022, 17, 911–923. 35995854 10.1038/s41565-022-01168-3

[8] C. Zhang , B. H. Wu , M. Q. Ma , Z. Wang , Z. K. Xu , Chem. Soc. Rev. 2019, 48, 3811–3841. 31179451 10.1039/c9cs00322c

[9] Y. Zhi , Z. Wang , H. L. Zhang , Q. Zhang , Small 2020, 16, 2001070. 10.1002/smll.202001070 32419332

[10] S. Daliran , A. R. Oveisi , Y. Peng , A. López‐Magano , M. Khajeh , R. Mas‐Ballesté , J. Alemán , R. Luque , H. Garcia , Chem. Soc. Rev. 2022, 51, 7810. 35938695 10.1039/d1cs00976a

[11] Y. Yang , H. Zhang , Y. Wang , L. Shao , L. Fang , H. Dong , M. Lu , L. Dong , Y. Lan , F. Zhang , Adv. Mater. 2023, 35, 2304170. 10.1002/adma.202304170 37363880

[12] A. E. Lakraychi , E. S. Picton , Y. Liang , D. L. Shaffer , Y. Yao , ACS Energy Lett. 2023, 8, 5032–5040.

[13] B. Hu , J. Xu , Z. Fan , C. Xu , S. Han , J. Zhang , L. Ma , B. Ding , Z. Zhuang , Q. Kang , X. Zhang , Adv. Energy Mater. 2023, 13, 2203540.

[14] J. Li , X. C. Jing , Q. Q. Li , S. W. Li , X. Gao , X. Feng , B. Wang , Chem. Soc. Rev. 2020, 49, 3565. 32369058 10.1039/d0cs00017e

[15] R. Iqbal , G. Yasin , M. Hamza , S. Ibraheem , B. Ullah , A. Saleem , S. Ali , S. Hussain , T. Anh Nguyen , Y. Slimani , R. Pathak , Coord. Chem. Rev. 2021, 447, 214152.

[16] R. Liu , K. T. Tan , Y. Gong , Y. Chen , Z. Li , S. Xie , T. He , Z. Lu , H. Yang , D. Jiang , Chem. Soc. Rev. 2021, 50, 120–242. 33283811 10.1039/d0cs00620c

[17] J. L. Wang , S. T. Zhuang , Coord. Chem. Rev. 2019, 400, 213046.

[18] X. H. Xiong , Z. W. Yu , L. L. Gong , Y. Tao , Z. Gao , L. Wang , W. H. Yin , L. X. Yang , F. Luo , Adv. Sci. 2019, 6, 1900547. 10.1002/advs.201900547 PMC6702651 31453066

[19] Y. Duan , Y. Yu , P. Liu , Y. Gao , X. Dai , L. Zhang , L. Chen , Y. Chen , Angew. Chem. Int. Ed. 2023, 62, 202302146. 10.1002/anie.202302146 36894504

[20] S. Bhunia , M. K. Jaiswal , K. A. Singh , K. A. Deo , A. K. Gaharwar , Adv. Healthcare Mater. 2022, 11, 2101737. 10.1002/adhm.202101737 PMC9354911 35104392

[21] J. Liang , J. Ruan , B. Njegic , A. Rawal , J. Scott , J. Xu , C. Boyer , K. Liang , Angew. Chem. Int. Ed. 2023, 62, e202303001. 10.1002/anie.202303001 37019840

[22] K. Dey , S. Mohata , R. Banerjee , ACS Nano 2021, 15, 12723.

[23] S. Kandambeth , K. Dey , R. Banerjee , J. Am. Chem. Soc. 2019, 141, 1807–1822. 30485740 10.1021/jacs.8b10334

[24] H. S. Sasmal , A. Kumar Mahato , P. Majumder , R. Banerjee , J. Am. Chem. Soc. 2022, 144, 11482. 35754375 10.1021/jacs.2c02301

[25] D. Rodríguez‐San‐Miguel , F. Zamora , Chem. Soc. Rev. 2019, 48, 4375–4386. 31310256 10.1039/c9cs00258h

[26] H. Wang , Z. T. Zeng , P. Xu , L. S. Li , G. M. Zeng , R. Xiao , Z. Y. Tang , D. L. Huang , L. Tang , C. Lai , D. N. Jiang , Y. Liu , H. Yi , L. Qin , S. J. Ye , X. Y. Ren , W. W. Tang , Chem. Soc. Rev. 2019, 48, 488. 30565610 10.1039/c8cs00376a

[27] K. Dey , M. Pal , K. C. Rout , S. Kunjattu H , A. Das , R. Mukherjee , U. K. Kharul , R. Banerjee , J. Am. Chem. Soc. 2017, 139, 13083–13091. 28876060 10.1021/jacs.7b06640

[28] H. Fan , J. Gu , H. Meng , A. Knebel , J. Caro , Angew. Chem. Int. Ed. 2018, 57, 4083–4087. 10.1002/anie.201712816 29405529

[29] B. Hosseini Monjezi , K. Kutonova , M. Tsotsalas , S. Henke , A. Knebel , Angew. Chem. Int. Ed. 2021, 60, 15153–15164. 10.1002/anie.202015790 PMC8359388 33332695

[30] X. Li , Z. Jia , J. Zhang , Y. Zou , B. Jiang , Y. Zhang , K. Shu , N. Liu , Y. Li , L. Ma , Chem. Mater. 2022, 34, 11062–11071.

[31] Z. Zhang , X. Shi , X. Wang , Z. Zhang , Y. Wang , Sep. Purif. Technol. 2023, 309, 123108.

[32] A. K. Mohammed , S. Usgaonkar , F. Kanheerampockil , S. Karak , A. Halder , M. Tharkar , M. Addicoat , T. G. Ajithkumar , R. Banerjee , J. Am. Chem. Soc. 2020, 142, 8252–8261. 32279483 10.1021/jacs.0c00555

[33] L. Huang , J. Yang , Y. Zhao , H. Miyata , M. Han , Q. Shuai , Y. Yamauchi , Chem. Mater. 2023, 35, 2661.

[34] S. Wang , L. Yang , K. Xu , H. Chen , N. Huang , ACS Appl. Mater. Interfaces 2021, 13, 44806–44813. 34519198 10.1021/acsami.1c14420

[35] S. Karak , K. Dey , R. Banerjee , Adv. Mater. 2022, 34, 2202751. 10.1002/adma.202202751 35760553

[36] F. Li , L.‐G. Ding , B.‐J. Yao , N. Huang , J.‐T. Li , Q. I.‐J. Fu , Y.U.‐B. Dong , J. Mater. Chem. A 2018, 6, 11140.

[37] L. G. Ding , B. J. Yao , F. Li , S. C. Shi , N. Huang , H. B. Yin , Q. Guan , Y. B. Dong , J. Mater. Chem. A 2019, 7, 4689–4698.

[38] Z. Huang , Y. H. Luo , W. Y. Geng , Y. Wan , S. Li , C. S. Lee , Small Methods 2021, 5, 2100036. 10.1002/smtd.202100036 34928098

[39] C. X. Li , J. Yang , P. Pachfule , S. Li , M. Y. Ye , J. Schmidt , A. Thomas , Nat. Commun. 2020, 11, 4712. 32948768 10.1038/s41467-020-18427-3 PMC7501297

[40] C. Ding , Y. Du , S. Agarwal , Adv. Funct. Mater. 2024, 34, 2309938.

[41] W. Li , W. Jin , H. Jiang , R. Wang , H. Jia , J. Liu , A. Tang , L. Zhu , D. Kong , Chem. Eng. J. 2023, 455, 140900.

[42] M. Zhang , L. Li , Q. Lin , M. Tang , Y. Wu , C. Ke , J. Am. Chem. Soc. 2019, 141, 5154–5158. 30912659 10.1021/jacs.9b01561

[43] S. Royuela , S. Sevim , G. Hernanz , D. Rodríguez‐San‐Miguel , P. Fischer , C. Franco , S. Pané , J. Puigmartí‐Luis , F. Zamora , Adv. Funct. Mater. 2024, 34, 2314634.

[44] S. Karak , K. Dey , A. Torris , A. Halder , S. Bera , F. Kanheerampockil , R. Banerjee , J. Am. Chem. Soc. 2019, 141, 7572–7581. 31017396 10.1021/jacs.9b02706

[45] J. Á. Martín‐Illán , D. Rodríguez‐San‐Miguel , O. Castillo , G. Beobide , J. Perez‐Carvajal , I. Imaz , D. Maspoch , F. Zamora , Angew. Chem. Int. Ed. 2021, 60, 13969–13977. 10.1002/anie.202100881 33724656

[46] F. Hu , Z. Hu , Y. U. Liu , K. Tam , R. Liang , Q. Xie , Z. Fan , C. Pan , J. Tang , G. Yu , W. Zhang , J. Am. Chem. Soc. 2023, 145, 27718–27727. 38083846 10.1021/jacs.3c10053

[47] R. Liu , Q. Yan , Y. Tang , R. Liu , L. Huang , Q. Shuai , J. Hazard. Mater. 2022, 421, 126702. 34325291 10.1016/j.jhazmat.2021.126702

[48] X. Zhao , P. Pachfule , S. Li , T. Langenhahn , M. Ye , C. Schlesiger , S. Praetz , J. Schmidt , A. Thomas , J. Am. Chem. Soc. 2019, 141, 6623. 30916950 10.1021/jacs.9b01226

[49] J. Á. Martín‐Illán , J. A. Suárez , J. Gómez‐Herrero , P. Ares , D. Gallego‐Fuente , Y. Cheng , D. Zhao , D. Maspoch , F. Zamora , Adv. Sci. 2022, 9, 2104643. 10.1002/advs.202104643 PMC8895050 35038248

[50] Q. Ma , L. Zeng , X. Liu , Q. Zhuang , J. Qian , Microporous Mesoporous Mater. 2022, 331, 111623.

[51] W. Zhao , T.‐P. Wang , J.‐L. Wu , R.‐P. Pan , X.‐Y. Liu , X.‐K. Liu , Chinese. J. Polym. Sci. 2019, 37, 1045–1052.

[52] C. Ding , M. Breunig , J. Timm , R. Marschall , J. Senker , S. Agarwal , Adv. Funct. Mater. 2021, 31, 2106507.

[53] P. Pachfule , S. Kandmabeth , A. Mallick , R. Banerjee , Chem. Commun. 2015, 51, 11717. 10.1039/c5cc04130a 26104390

[54] K. Xiong , Y. Wang , F. Zhang , X. Li , X. Lang , Appl. Catal. B 2023, 322, 122135.

[55] S. Kandambeth , A. Mallick , B. Lukose , M. V. Mane , T. Heine , R. Banerjee , J. Am. Chem. Soc. 2012, 134, 19524–19527. 23153356 10.1021/ja308278w

[56] J. Zhang , C. Cheng , L. Guan , H. L. Jiang , S. Jin , J. Am. Chem. Soc. 2023, 145, 21974–21982. 37779433 10.1021/jacs.3c06764

[57] M. Thommes , K. Kaneko , A. V. Neimark , J. P. Olivier , F. Rodriguez‐Reinoso , J. Rouquerol , K. S. Sing , Pure Appl. Chem. 2015, 87, 1051.

[58] W. He , K. Kong , M. Wang , B. Dong , D. Yuan , K. P. Bryliakov , R. Wang , Appl. Catal. B 2024, 350, 123916.

[59] S. Jiang , S. Agarwal , A. Greiner , Angew. Chem. Int. Ed. 2017, 56, 15520. 10.1002/anie.201700684 28621026

[60] H. Hu , Z. Zhao , W. Wan , Y. Gogotsi , J. Qiu , Adv. Mater. 2013, 25, 2219–2223. 23418081 10.1002/adma.201204530

[61] D. Zhu , Y. Zhu , Q. Yan , M. Barnes , F. Liu , P. Yu , C. Tseng , N. Tjahjono , P. Huang , M. M. Rahman , E. Egap , P. M. Ajayan , R. Verduzco , Chem. Mater. 2021, 33, 4216–4224.

[62] Q. Wang , P. Wang , Y. Wang , Y. Xu , H. Xu , K. Xi , ACS Appl. Mater. Interfaces 2024, 16, 37052–37062. 38965714 10.1021/acsami.4c07017

[63] X. Liu , G. J. H. Lim , Y. Wang , L. Zhang , D. Mullangi , Y. Wu , D. Zhao , J. Ding , A. K. Cheetham , J. Wang , Chem. Eng. J. 2021, 403, 126333.

[64] F. Monehzadeh , Z. Rafiee , Appl. Organomet. Chem. 2020, 34, 5631.

[65] X. Hu , Z. Zhan , J. Zhang , I. Hussain , B. Tan , Nat. Commun. 2021, 12, 6596. 34782615 10.1038/s41467-021-26817-4 PMC8593010

[66] S. Cao , D. Li , A. A. Uliana , Y. Jiang , J. Zhu , Y. Zhang , B. Van der Bruggen , Appl. Catal., B 2023, 323, 122175.

