# Supplementary material for: Ultra‐Low Density Covalent Organic Framework Sponges with Exceptional Compression and Functional Performance
Source: Angew Chem Int Ed Engl. 2025 Mar 27;64(22):e202502513. doi: 10.1002/anie.202502513 (PMC12105692; doi:10.1002/anie.202502513)
Supplement: Supplementary file 1 — Supporting Information [file ANIE-64-e202502513-s003.docx]

Supporting Information

Ultra-Low Density Covalent Organic Framework Sponges with Exceptional Compression and Functional Performance

*Chenhui Ding,^[a]^ Yingying Du,^[a]^ Tamara Fischer,^[b]^ Jürgen Senker,^[b]^ Seema Agarwal*^[a]^*

[a] C. Ding, Y. Du, Prof. S. Agarwal

Macromolecular Chemistry and Bavarian Polymer Institute

University of Bayreuth

Universitätsstrasse 30, 95440 Bayreuth, Germany

E-mail: agarwal@uni-bayreuth.de

[b] T. Fischer, Prof. J. Senker

Department of Chemistry, Inorganic Chemistry III, and Northern Bavarian NMR Centre

University of Bayreuth

Universitätsstrasse 30, 95440 Bayreuth, Germany

**Experimental Section**

*Materials*

Polyacrylonitrile (PAN, Mw = 80,000, Carl Roth). 1,3,5-triformylphloroglucinol (Tp), p-phenylenediamine (Pa), 2,5-dimethyl-1,4-phenylenediamine (Pa-(CH_3_)_2_), benzidine (BD), [2,2'-bipyridine]-5,5'-diamine (Bpy), malononitrile, benzaldehyde, and ethyl cyanoacetate were bought from Sigma-Aldrich. 3,5-di-tert-butyl-2-hydroxybenzaldehyde, 4-bromobenzaldehyde, 4-methylbenzaldehyde, [1,1'-biphenyl]-4-carbaldehyde, and ethyl cyanoformate were supply by [Acros Organics. Dodecane and anthracene-9-carbaldehyde were brought by Alfa Aesar.](https://www.thermofisher.com/de/de/home/chemicals/acros-organics.html) All organic solvents were from Fisher Chemical.

*Preparation of the pure COF sponges*

A three-step preparation method was used to make pure COF sponges. Here, a procedure is described as an example for making TpPa sponge-3. In the first step, 18 mg of Pa was dissolved in 6 mL (PAN was 60 mg) of PAN/DMSO (dimethyl sulfoxide) solution, and PAN/Pa sponge was obtained by freeze-drying. In the second step, 78 mg of PAN/Pa sponge (Pa was 18 mg, 0.166 mmol) was added to Tp (23.3 mg, 0.111 mmol), and acetic acid (0.23 g) dichloromethane (11.5 g) solution. The reaction was done at 120 °C for 1 day to obtain a red PAN/TpPa sponge. It was washed with dichloromethane and acetone at least three times, and vacuum dried for 12-16h at 60 °C. In the third step, the template PAN was removed from PAN/TpPa sponge by Soxhlet extraction for 1 day in dimethylformamide (DMF). And after washing it three times with deionized water, freeze-drying gave the resultant TpPa sponge. For the preparation of TpPa sponge-5 and TpPa sponge-10 sponges, the same procedure was used; only the amounts of starting Pa were different. 30 mg and 60 mg of Pa are dissolved in a 6 mL solution of PAN in DMSO (PAN was 60 mg) for making TpPa sponge-5 and TpPa sponge-10 sponges, respectively. For making TpPa sponge-20, 20 mg of Pa is dissolved in 6 ml solution of PAN in DMSO (PAN was 120 mg).

The other three imine-linked COFs (TpPa-(CH_3_)_2_, TpBD, and TpBpy) sponges were prepared using the same method, with only the first step modified appropriately. 60 mg of Pa-(CH_3_)_2_, BD, and Bpy were dissolved in 6 mL solution of PAN in DMSO (60 mg of PAN), respectively, and the other steps were the same, and the resulting pure COF sponges were named TpPa-(CH_3_)_2_ sponge, TpBD sponge and, TpBpy sponge, respectively.

In this work, the COF sponges were obtained in a cylindrical form. Their mass (m) was determined by weighing, and the density (ρ, mg cm⁻³) was calculated using the following formula:

$$\rho=\frac{m}{V}=\frac{m}{h\cdot d^{2}\cdot\pi\cdot\frac{1}{4}}$$

where m (mg) is the mass, h (cm) is the height, and d (cm) is the diameter of the cylindrical COF sponge.

*Synthesis of TpPa powder*

Tp (23.3 mg, 0.111 mmol) and Pa (18 mg, 0.166 mmol) reacted at 120^o^C in dichloromethane (11.5 g) having acetic acid (0.23 g) for 1 day to obtain. The resulting TpPa red powder was washed with dichloromethane and acetone several times, and finally dried in vacuum oven for about 12-14 h at 60 °C.

**Characterization and instruments**

In a freeze dryer, the cooling was from room temperature to -50 °C with 5 °C min^−1^ cooling rate for 60 min, followed by bringing the contents to 20 °C. The final drying was done at 0.25 mbar for 24 h to obtain various sponges.

Scanning electron microscopy (SEM): FEI Quanta FEG 250 (voltage 10 kV, Everhart-Thornley secondary electron detector) was used. A Sputter Coater 208HR, Cressington, was used for sputtering the samples with 1.8 nm layer of platinum before imaging the samples.

Optical microscope: Optical microscope images and videos of all samples were provided by ZEISS Smartzoom 5.

Fourier transform infrared (FT-IR): FT-IR spectra were recorded using Digilab Excalibur FTS-3000 between 600-4000 cm^−1^.

^13^C and ^15^N solid-state NMR spectroscopy: Bruker Avance III HD spectrometer operating at a B_0_ field of 9.4 T (ν_0_(^13^C) = 100.6 MHz and ν_0_(^15^N) = 40.6 MHz) was used for^13^C and ^15^N solid-state NMRs. The samples were spun at 15.0 kHz (^13^C) and 10 kHz (^15^N) in a 3.2 mm MAS triple resonance probe. ^13^C MAS spectra were obtained with ramped cross-polarization (CP) experiments where the ^13^C nutation was set to 50 kHz and the ^1^H nutation frequency ν_nut_ was varied linearly from 50‑100% to match the Hartmann‑Hahn conditions. The contact time was set to 3 ms. The ^15^N CP MAS NMR spectra were acquired with a proton ramp (70‑100%) with a contact time of 5 ms where the ^15^N nutation was set to 21 kHz. Proton broadband decoupling with spinal-64 and ν_nut_ = 70 kHz was applied during acquisition for both the ^13^C and ^15^N MAS NMR spectra. The spectra are referenced with respect to tetramethylsilane (^13^C) and CH_3_NO_2_ (^15^N).

Bragg-Brentano type diffractometer (XPERT-PRO, PANalytical B.V.) with Cu Kα radiation (λ = 1.540598 Å) was used for XRD patterns. The measuring range of each sample is 2θ = 2° to 40° at a rate of 1° min^−1^.

N_2_ adsorption-desorption isotherms: Micromeritics ASAP 2020 HD88 instrument was employed for N_2_ adsorption-desorption isotherms. The samples were degassed under vacuum at 120 °C for 12 h before measurement. The surface area, pore size distribution, and cumulative pore volume were calculated by Brunauer-Emmett-Teller (BET) method and nonlocal density functional theory (NLDFT) model.

Mechanical properties: A tensile tester (ZwickiLine Z0.5; BT1-FR0.5TN.D14; Zwick/Roell, Germany) was used for compression testing and fatigue testing of the TpPa sponges (cylinders with a diameter of 1.8 cm and a height of 2 cm ) at a compression rate of 100 mm min^−1^.

The instrument used for GC measurements was GC-FID system (GC-2010 Plus, Shimadzu). The carrier gas was nitrogen. For measurements, 10 µL of the test contents were dissolved in 1 mL acetonitrile. 1 µL of this acetonitrile-test content solution is injected (split ratio 1:50). The temperature measurement range was from 50 °C to 300 °C with a heating rate of 15 K min^−1^. The temperature at 50^o^C was hold for 2 min.

**Methods**

*Absorption measurement*

Drying of samples at 60 °C in a vacuum for 6 h was done before any measurement. The gravimetric method was used after immersing the samples for 5 minutes, each in various organic solvents at room temperature. The weight of the sample before absorption (*W_before_*, g) and after absorption (*W_after_*, g) was recorded, and the absorption capacity (*Q*) of the samples was calculated according to the following formula (1).

$Q=\frac{W_{after}-W_{before}}{W_{before}}\times100\%$ (1)

Most organic solvents can be quickly desorbed by simply squeezing the samples, and chloroform is repeatedly absorbed for recyclability testing. After the absorption measurement, the absorbed solvent was squeezed out and washed a few times with acetone and deionized water, respectively, and the dried sponge samples obtained after freeze-drying were used again for reusability tests.

*Catalyst activity test*

Typically, 2.5 mmol of various substituted benzaldehydes, 5.0 mmol methylene compounds, 6.8 mg catalyst (TpPa sponges or TpPa powder), and 10 mL DMF were taken in an appropriate-size reaction tube. 0.75 mmol dodecane was used as an internal standard in this reaction mixture. Let the reaction proceed for different times at 80 °C. The reaction contents were continuously stirred at 200 rpm. The products of the reaction were determined by GC/MS, and the product yields were calculated by the GC-FID system (GC-2010 Plus, Shimadzu). After the reaction, the TpPa sponge was directly taken out of the reaction medium and washed five times with fresh DMF, and finally most of the DMF was expelled by squeezing and reused for recycle runs. After the catalytic experiment, theTpPa sponges were washed as described in the absorption test section and then again used for subsequent recyclability tests.

**Supplementary figures and tables**


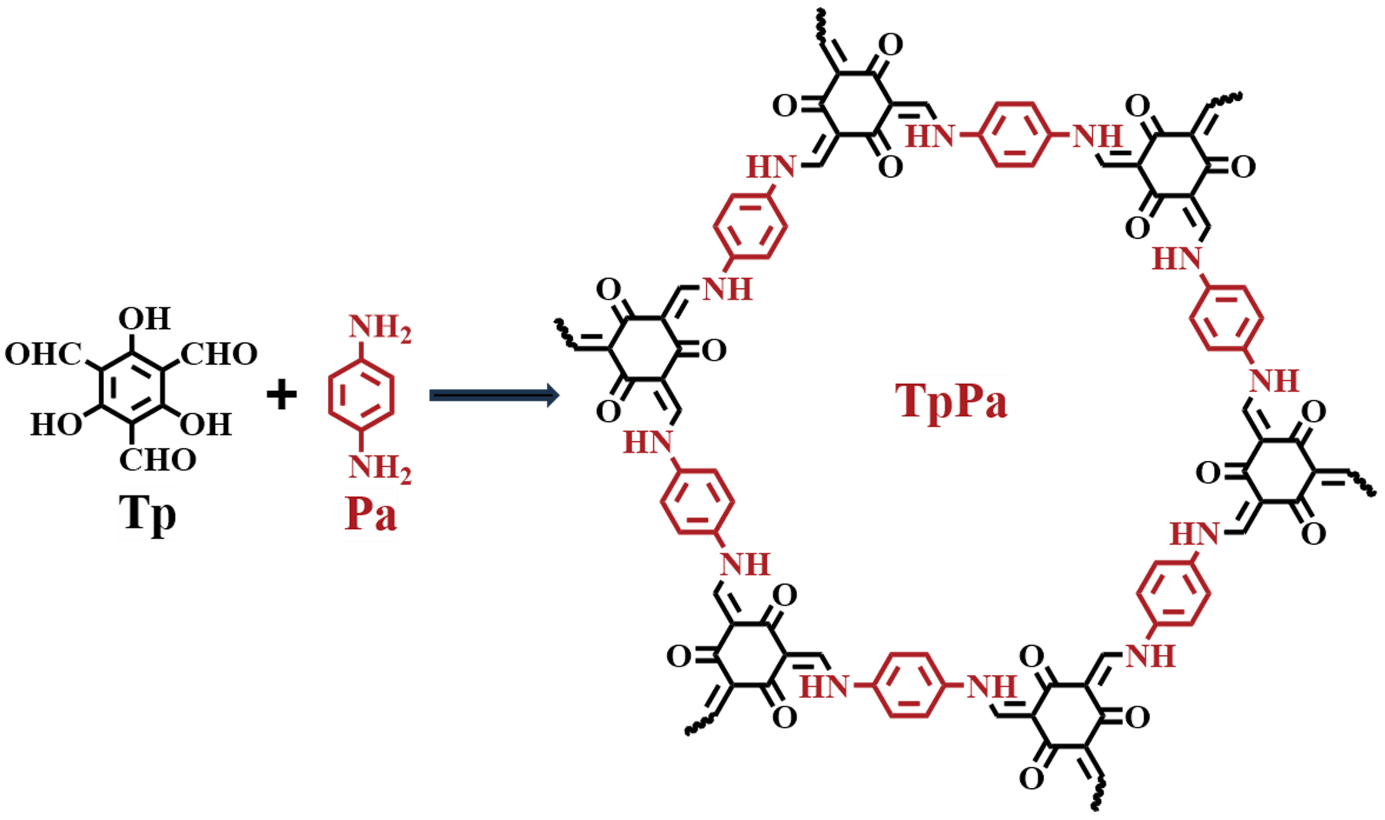


**Figure S1.** Synthetic reaction scheme showing the formation of TpPa.


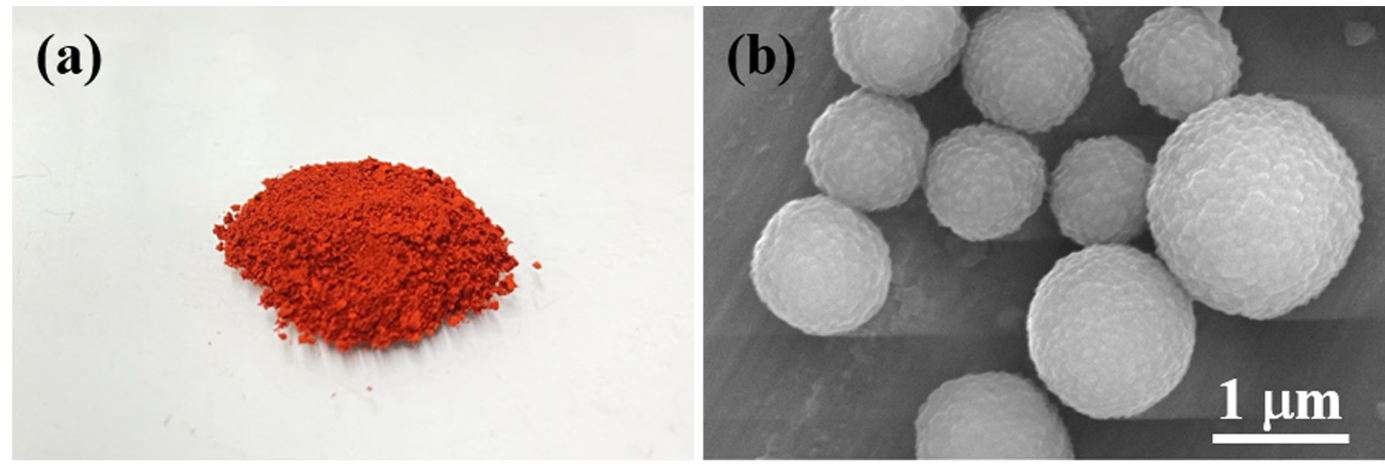


**Figure S2.** Photograph (a) and SEM image (b) of TpPa powder.

**Table S1.** The pore size, skeleton thickness and density of TpPa sponges.

| TpPa sponges | Pore size of hierarchical  porous structure  [μm] | The thickness of the TpPa skeleton  [nm] | Density  [mg cm^−3^] |
| --- | --- | --- | --- |
| TpPa sponge-3 | 211.1±92.9 | 103.1±8.7 | 2.2±0.1 |
| TpPa sponge-5 | 217.2±106.1 | 168.4±12.3 | 3.6±0.2 |
| TpPa sponge-10 | 202.7±91.5 | 213.8±19.1 | 6.7±0.4 |
| TpPa sponge-20 | 124.3±52.2 | 257.4±21.5 | 12.3±0.7 |


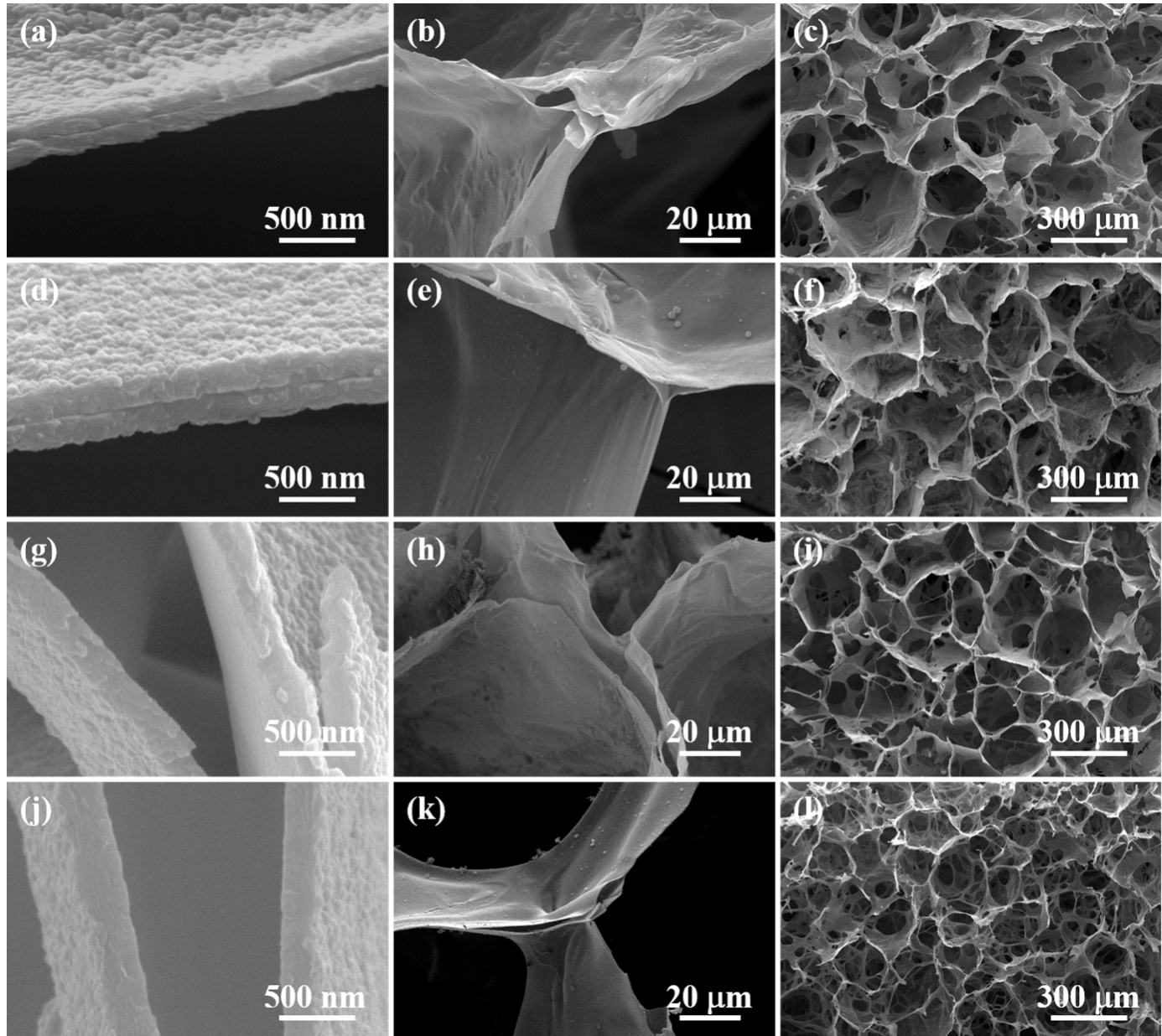


**Figure S3.** SEM images of TpPa sponge-3 (a-c), TpPa sponge-5 (d-f), TpPa sponge-10 (g-i), and TpPa sponge-20 (j-l), illustrating their microstructures at varying levels of magnification.

**Table S2.** The density of TpPa sponges in this work were compared with other reported COF monolithic materials.

| Materials | Density  [mg cm^−3^] | Reference |
| --- | --- | --- |
| **TpPa sponge-3** | **2.2** | **This work** |
| **TpPa sponge-5** | **3.6** |  |
| **TpPa sponge-10** | **6.7** |  |
| **TpPa sponge-20** | **12.3** |  |
| PCA_0.5_ | 4.5 | Ref.1 |
| COF/rGO aerogel | 7 | Ref.2 |
| CGA-4.6 | 7.1 | Ref.3 |
| MF@COF | 8.15 | Ref.4 |
| PI/COF-SO_3_H-0.5 | 11.2 | Ref.5 |
| COF/CS aerogel | 12.4 | Ref.6 |
| TpPa-1 FAG | 14.1 | Ref.7 |
| COF/cellulose hybrid aerogels | 15 | Ref.8 |
| Agel-COF[4 + 3]_2_-CHO | 17.1 | Ref.9 |
| TAPB-PDA-AGCOF | 17.3 | Ref.10 |
| COFA-3 | 18.1 | Ref.11 |
| DT-COF aerogel | 18.43 | Ref.12 |
| OH−COF/Re-CP aerogel | 19.7 | Ref.13 |
| COF-Pa monolith | 20 | Ref.14 |
| TAPD-PDA | 26 | Ref.15 |
| TFPT-HZ-COF | 29 | Ref.16 |
| COF-IL@chitosan | 32 | Ref.17 |
| TAPB-TPA-COFs-aerogel | 46 | Ref.18 |
| COF-chitosan aerogel | 56 | Ref.19 |
| COFs-aerogel | 59 | Ref.20 |
| NKCOF-12 | 230 | Ref.21 |


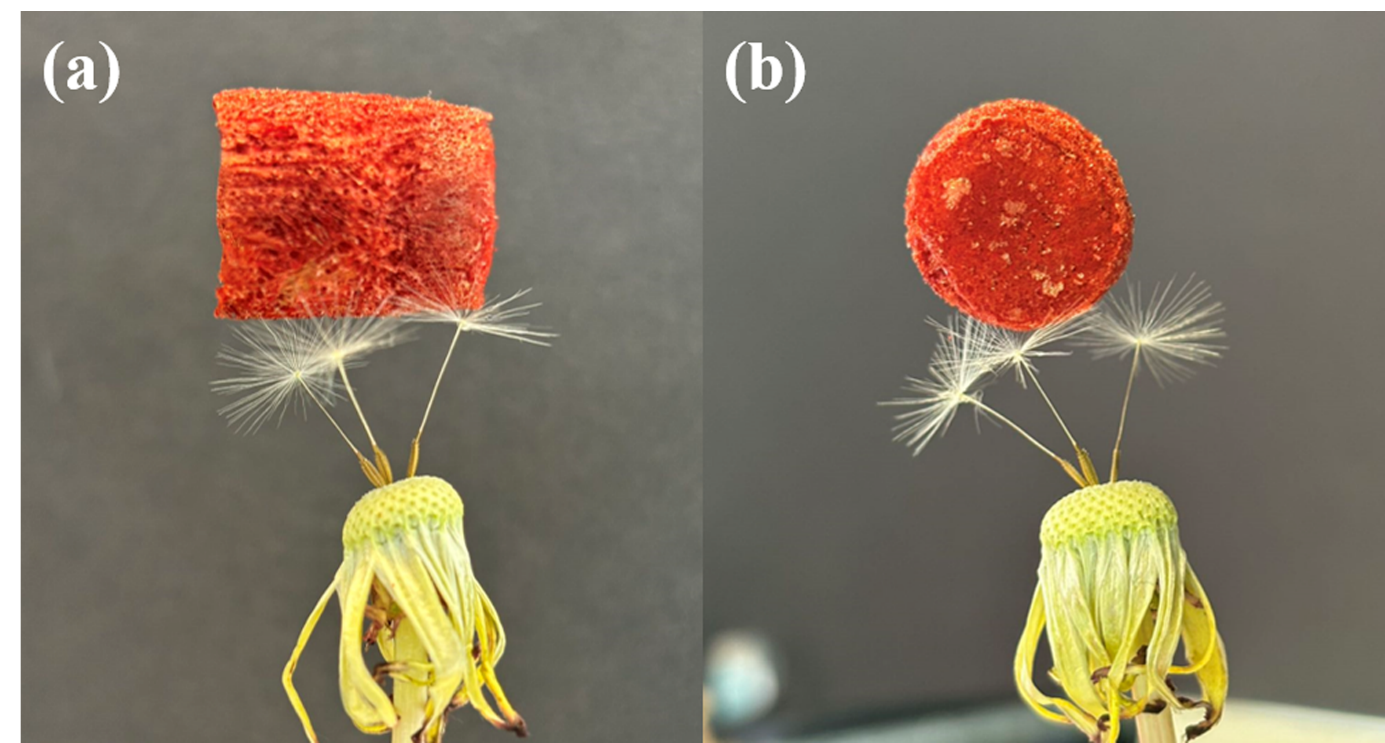


**Figure S4.** Photographs of TpPa sponge-3 (5.8 mg) showcasing its remarkable lightness: (a) front view and (b) side view, as it stands balanced atop three dandelion hairs.


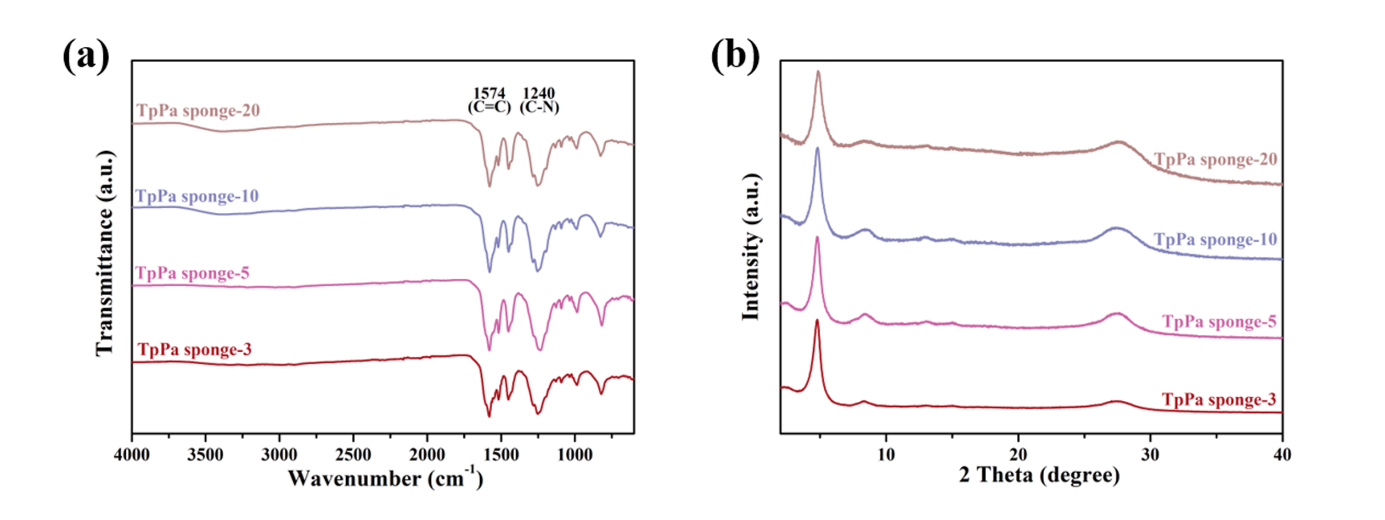


**Figure S5.** FT-IR spectra (a) and XRD patterns (b) of TpPa sponge-3, TpPa sponge-5, TpPa sponge-10, and TpPa sponge-20.


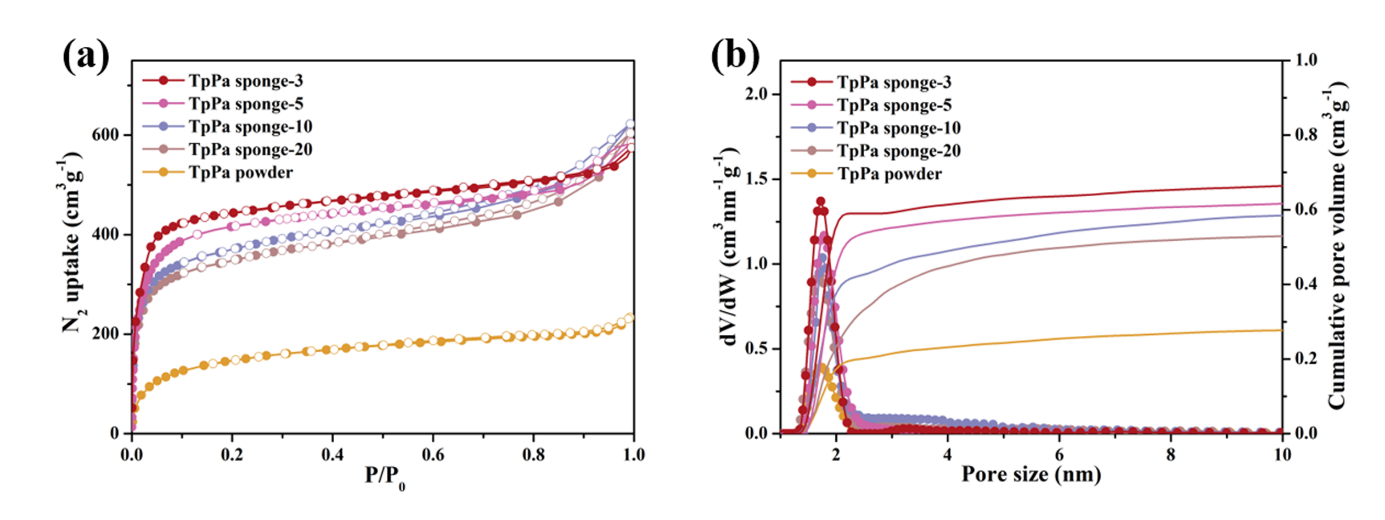
**Figure S6.** N_2_ adsorption-desorption isotherms (a) and the pore size distribution and cumulative pore volume of TpPa sponges and TpPa powder (b).

**Table S3.** BET surface area, pore size, and cumulative pore volume of TpPa sponges and TpPa powder.

| Materials | BET surface area  [m^2^ g^−1^] | Pore size  [nm] | Cumulative pore volume  [cm^3^ g^−1^] |
| --- | --- | --- | --- |
| TpPa powder | 561 | 1.75 | 0.28 |
| TpPa sponge-20 | 1359 | 1.72 | 0.53 |
| TpPa sponge-10 | 1480 | 1.81 | 0.58 |
| TpPa sponge-5 | 1528 | 1.78 | 0.62 |
| TpPa sponge-3 | 1655 | 1.84 | 0.67 |


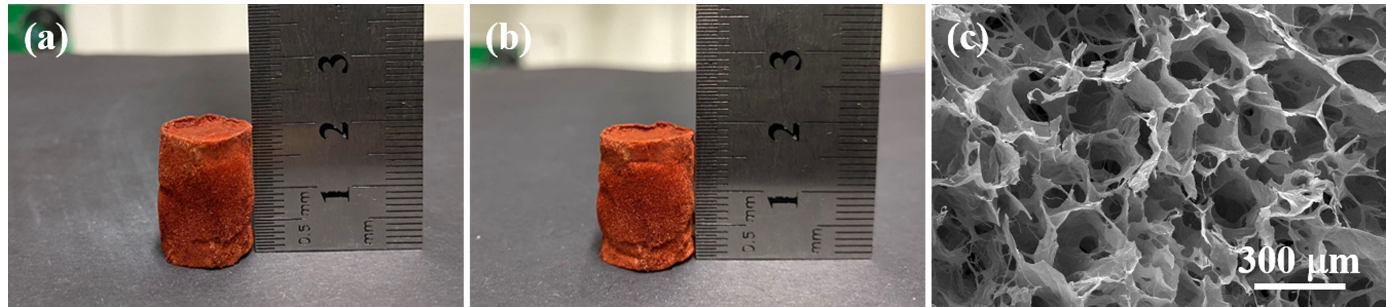


**Figure S7.** The photographs of the changes in the TpPa sponge-3 before (a) and after(b) 300,000 cycles of compressing it up to 50% strain, and SEM image of the interior after compression cycles (c).


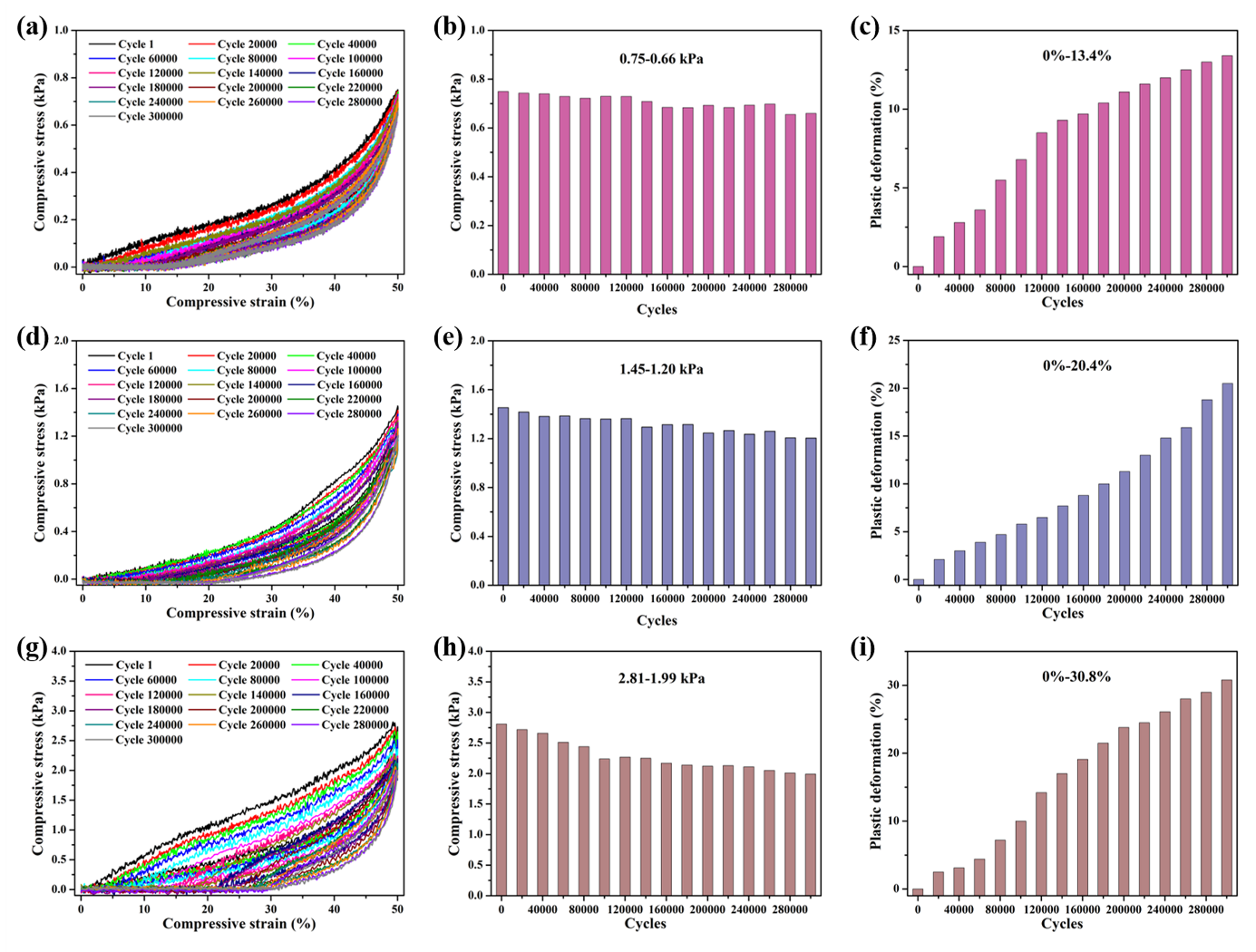


**Figure S8.** The stress-strain (compressive) curves at 50% strain (300,000 cycles) of TpPa sponge-5 (a-c), TpPa sponge-10 (d-f), and TpPa sponge-20 (g-i) for studying the cycle-dependent compressive stress, and cycle-dependent height reduction rate.

**Table S4.** The compressive stress and sponge height reduction rate of TpPa sponges after 300,000 compression cycles at 50% strain.

| Materials | Compression cycles | Compression stress reduction rate  [%] | Plastic deformation  [%] |
| --- | --- | --- | --- |
| TpPa sponge-3 | 300000 | 7.9 | 7.1 |
| TpPa sponge-5 | 300000 | 12 | 13.4 |
| TpPa sponge-10 | 300000 | 17.2 | 20.4 |
| TpPa sponge-20 | 300000 | 29.1 | 30.8 |


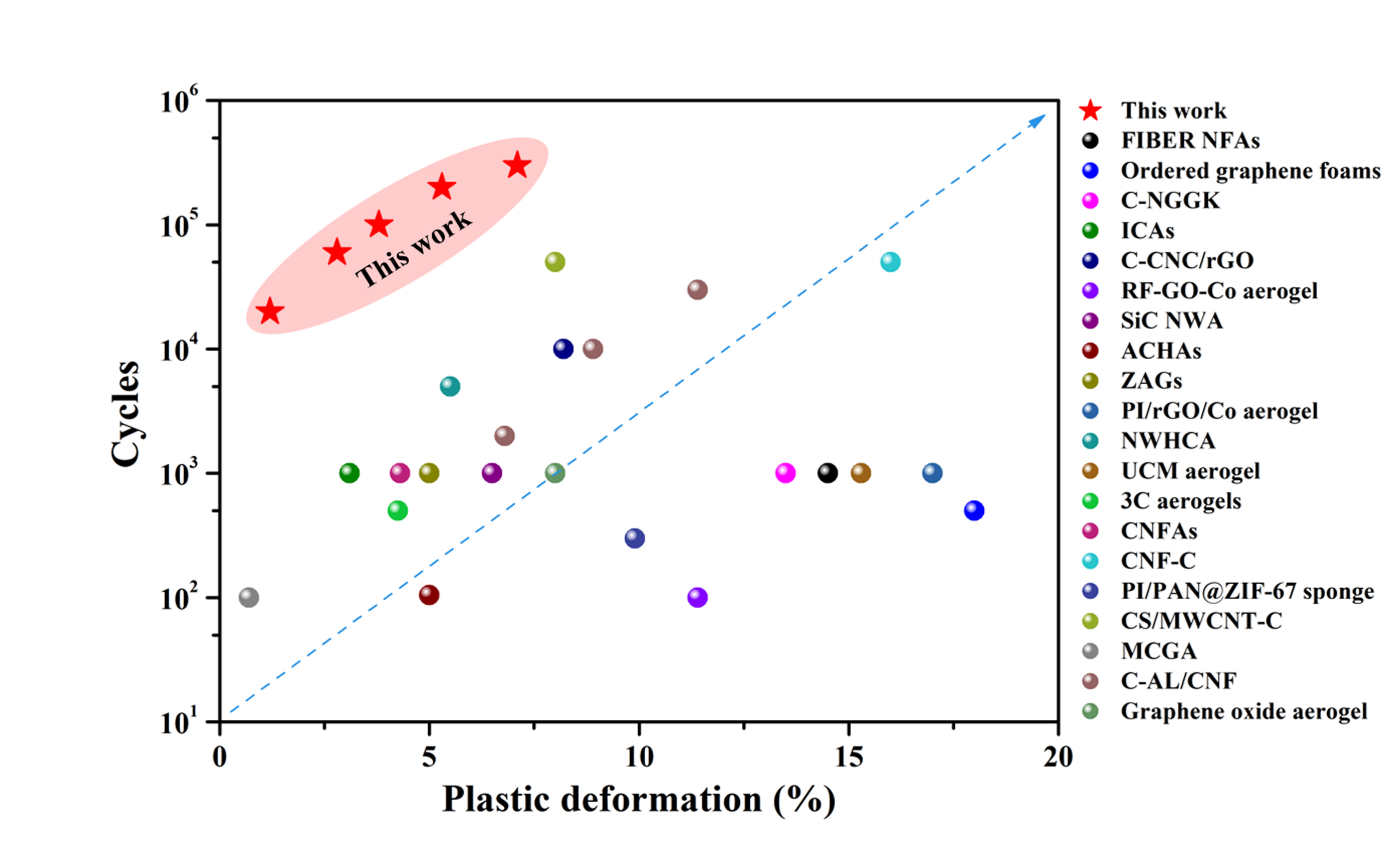


**Figure S9.** Comparison of fatigue resistance of TpPa sponge-3 and other 3D macroscopic objects (compression ratio of 50%-60%). The detailed information is shown in **Table S5**.

**Table S5.** Comparison of fatigue resistance of TpPa sponge-3 and other 3D macroscopic objects (compression ratio of 50%-60%).

| Materials | Plastic deformation  [%] | Compression  cycles | Compression strain [%] | Reference |
| --- | --- | --- | --- | --- |
| **TpPa sponge-3** | **1.2** | **20000** | **50** | **This work** |
|  | **2.8** | **60000** |  |  |
|  | **3.8** | **100000** |  |  |
|  | **5.3** | **200000** |  |  |
|  | **7.1** | **300000** |  |  |
| FIBER NFAs | 14.5 | 1000 | 60 | Ref.22 |
| Ordered graphene foams | 18 | 500 | 60 | Ref.23 |
| C-NGGK | 13.5 | 1000 | 50 | Ref.24 |
| ICAs | 3.1 | 1000 | 50 | Ref.25 |
| C-CNC/rGO | 8.2 | 10000 | 50 | Ref.26 |
| RF–GO–Co aerogel | 11.4 | 100 | 50 | Ref.27 |
| SiC NWA | 6.5 | 1000 | 60 | Ref.28 |
| ACHAs | 5 | 105 | 50 | Ref.29 |
| ZAGs | 5 | 1000 | 50 | Ref.30 |
| PI/rGO/Co aerogel | 17 | 1000 | 50 | Ref.31 |
| NWHCA | 5.5 | 5000 | 50 | Ref.32 |
| UCM aerogel | 15.3 | 1000 | 60 | Ref.33 |
| 3C aerogels | 4.25 | 500 | 50 | Ref.34 |
| CNFAs | 4.3 | 1000 | 50 | Ref.35 |
| CNF–C | 16 | 50000 | 50 | Ref.36 |
| PI/PAN@ZIF-67 sponge | 9.9 | 300 | 50 | Ref.37 |
| CS/MWCNT-C | 8 | 50000 | 50 | Ref.38 |
| MCGA | 0.7 | 100 | 50 | Ref.39 |
| C-AL/CNF-5 | 6.8 | 2000 | 50 | Ref.40 |
|  | 8.9 | 10000 | 50 |  |
|  | 11.4 | 30000 | 50 |  |
| Graphene oxide aerogel | 8 | 1000 | 50 | Ref.41 |


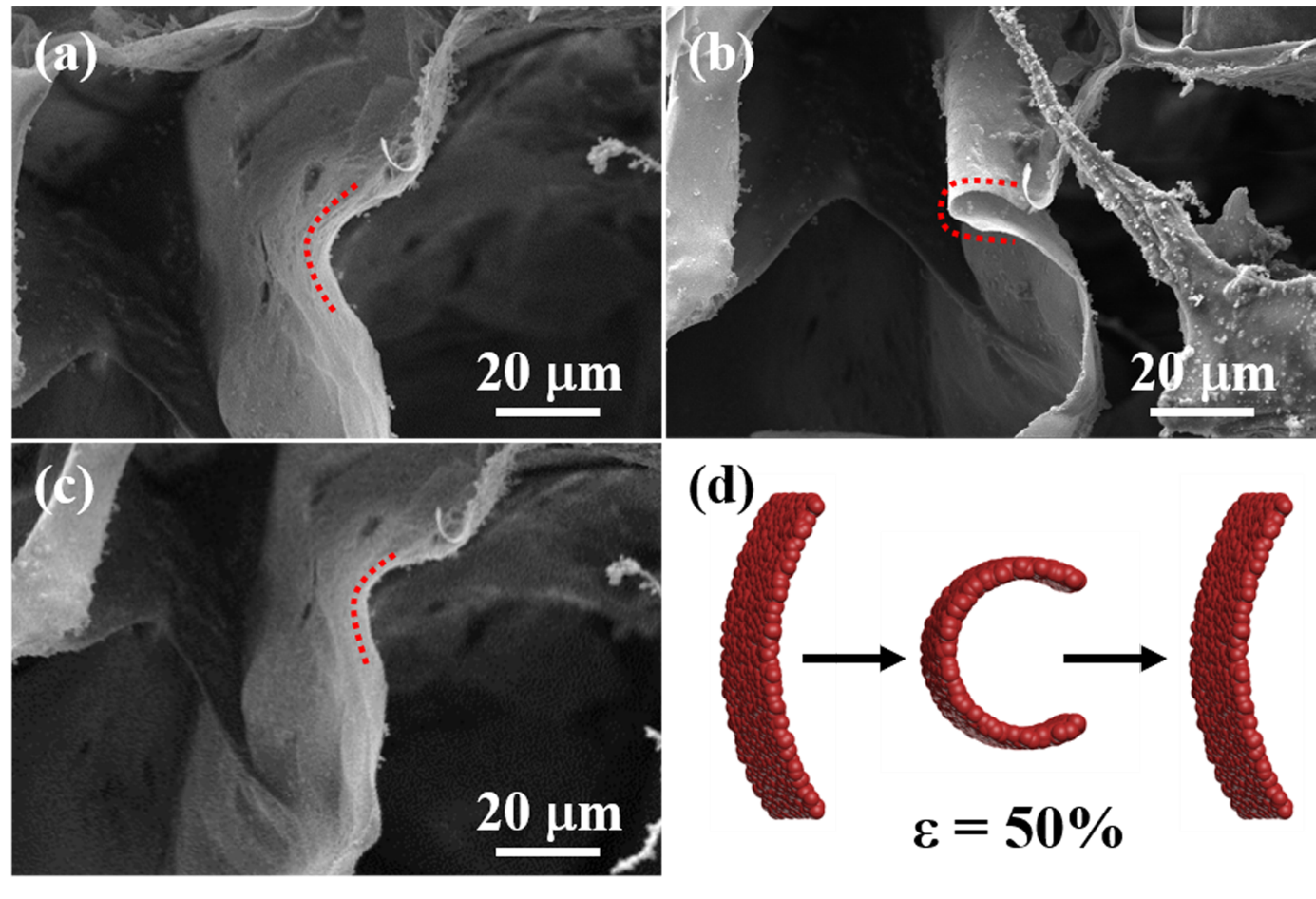


**Figure S10a.** SEM images of single TpPa sponge skeleton at different states: initial (a), 50% strain (b), and after stress release (c). Schematic diagram of the changes of single TpPa sponge skeleton at 50% strain deformation (d).


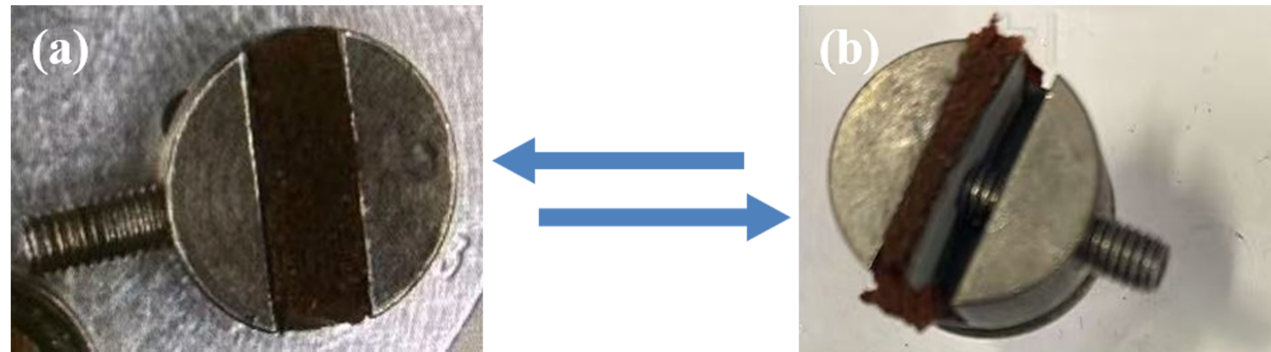


**Figure S10b.** The different compression states of the COF sponge in a special sample holder that is used for taking SEM images. (a) Pre- and post-compression state, (b) during compression.

The COF sponge was placed on a dedicated cross-sectional sample stage (Figure S10b, left, initial state) to capture the SEM image before compression. Subsequently, the sponge was compressed by 50% by rotating the screw to acquire SEM images in the compressed state. Finally, the sponge was returned to its non-compressed initial state by reversing the screw, and post-compression SEM image was taken.

**Table S6.** Comparison of absorption performance of TpPa sponge-3 with other absorbents

| Materials | Absorbate | Capacity  [g g^−1^] | Reference |
| --- | --- | --- | --- |
| **TpPa sponge-3** | **Ethanol, Acetone, Cyclohexane, Hexane, THF, Toluene, Methanol, Acetonitrile, DMF, Ethyl acetate, Dioxane, DMSO, Silicone oil, Chloroform** | **201-262** | **This work** |
| TpPa-1 FAG | DMSO, Chloroform, Toluene, Dioxane, Acetone, DMAc, Methanol, Ethanol | 43-90 | Ref.7 |
| TAPB-TFPA | Ethanol, DMAc, Methanol, DCB, Hexane, THF, Mesitylene, Acetone, Dioxane, Toluene, Chloroform, DMSO | 16-35 | Ref.15 |
| COFA-1 | DMSO, DMAc, DMF, THF, DCM, Chloroform, Acetonitrile, Hexane, Acetone, Ethanol, Methanol | 17-27 | Ref.11 |
| TMT-BPA-COF | Chloroform, Toluene, Rapeseed oil, DOX, DMSO, Acetone, THF, Methanol, DMF, Ethylene glycol, Silicone oil, Dichloromethane, Hexane | 2-5 | Ref.42 |
| TAPB-PDA-AGCOF | Toluene | 32.6 | Ref.10 |
| Agel-COF[4 + 3]_2_-CHO | DMF, Toluene, DCM, NMP, O-DCB, n-BuOH, Dioxane, DEAc, NEP, Ethanol, DEF, CTC, Acetone, THF, DMAc | 5-70 | Ref.9 |
| COF-DTF@foam | Phenoxin, Olive oil, Acetone, Dichloromethane, Petroleum ether, Hexane, Ethanol, Toluene, DMF, Chloroform | 69.7-173.2 | Ref.43 |
| COF-VF@foam | Chloroform, Nitrobenzene, DMF, Toluene, Bromobenzene, Ethanol, Hexane, Mineral oil, Pump oil, Soybean oil | 67-142 | Ref.44 |
| Sponges@COFs | Pump oil, Silicone oil, Rapeseed oil, Ethyl acetate, n-decane, Cyclohexane, Hexane, Toluene | 81-150 | Ref.45 |
| [CF_3_]-COF@sponge | Petroleum ether, Gasoline, Acetone, Toluene, Ethyl acetate, DMF, Dimethicone, Nitrobenzene, Dichloromethane, Trichloromethane, Tetrachloromethane | 34-73 | Ref.46 |
| DT-COF aerogel | Acetone, CTC, Toluene, DMSO, Mesitylene, THF, n-BuOH, Dioxane, DMF, DMAc, DCM, o-DCB, Ethanol | 20-40 | Ref.12 |
| COF/rGO | Hexane, Cyclohexane, Methanol, Toluene, DMSO, Silicone oil, Chloroform, Phenixin, Ethyl acetate, Acetone, DMF, Ethylene glycol, Dioxane, Ethanol, DMA, THF | 98‒240 | Ref.2 |
| Spongy graphene | Methanol, Ethanol, Acetone, THF, DMSO, Toluene, Ethylbenzene, 1,2-dichlorobenzene, Chloroform, Nitrobenzene, Hexane, Heptane, Octane, Decane, Dodecane, Pump oil, Kerosene, Caster oil, Soybean oil | 20-86 | Ref.47 |
| ZIF-8@rGO@Sponge | n-Heptane, Ethyl acetate, Dibromoethane, Butanone, Acetone, Toluene, Tetrachloromethane, Chloroform, Silicone oil, Bump oil, Bean oil | 14-37 | Ref.48 |
| CNT sponges | Hexane, Ethanol, Gasoline, Vegetable oil, Pump oil, Diesel oil, DMF, Ethylene glycol, Chloroform | 80-180 | Ref.49 |
| Graphene-CNT hybrid foam | Compressor oil, Sesame oil, Chloroform, Dichlorobenzene, Toluene, DMF | 80-130 | Ref.50 |


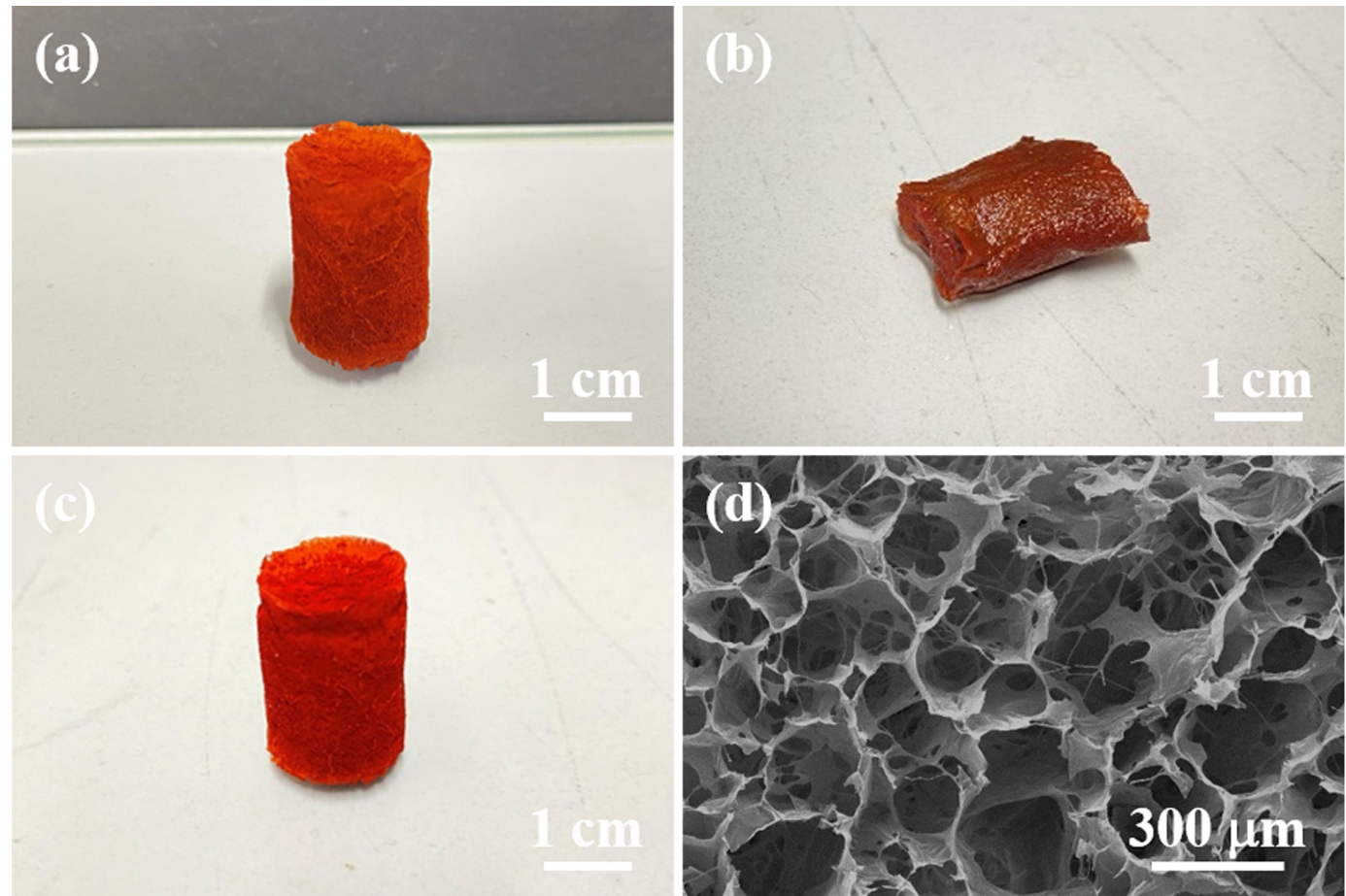


**Figure S11.** Photographs of TpPa sponge-3 before (a), during (b), and after(c) absorbing organic solvents. SEM iamge of TpPa sponge-3 after 20 cycles of organic solvent absorption (d).


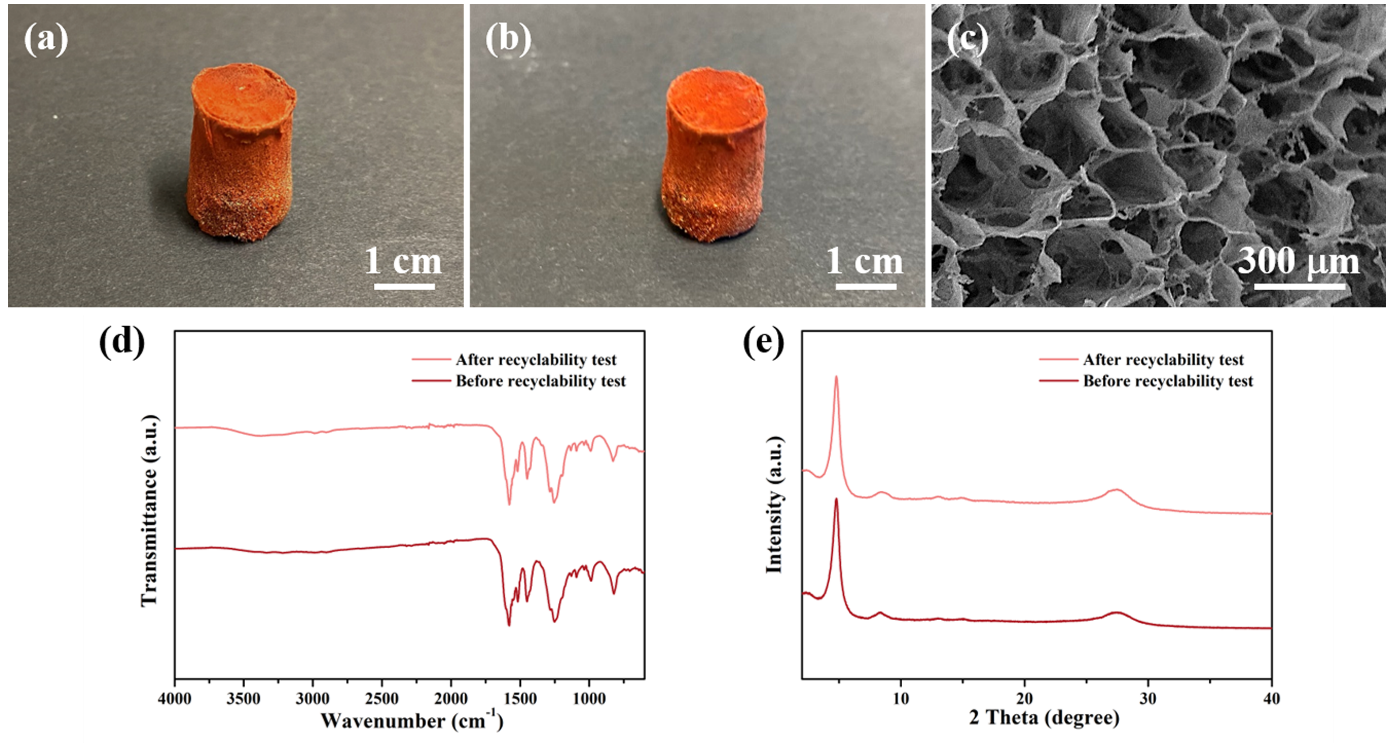


**Figure S12.** Photographs of TpPa sponge-3 before (a) and after (b) recyclability test. SEM image of TpPa sponge-3 after the recyclability test (c). FT-IR spectra (d) and PXRD patterns (e) of TpPa sponge-3 before and after recyclability test.

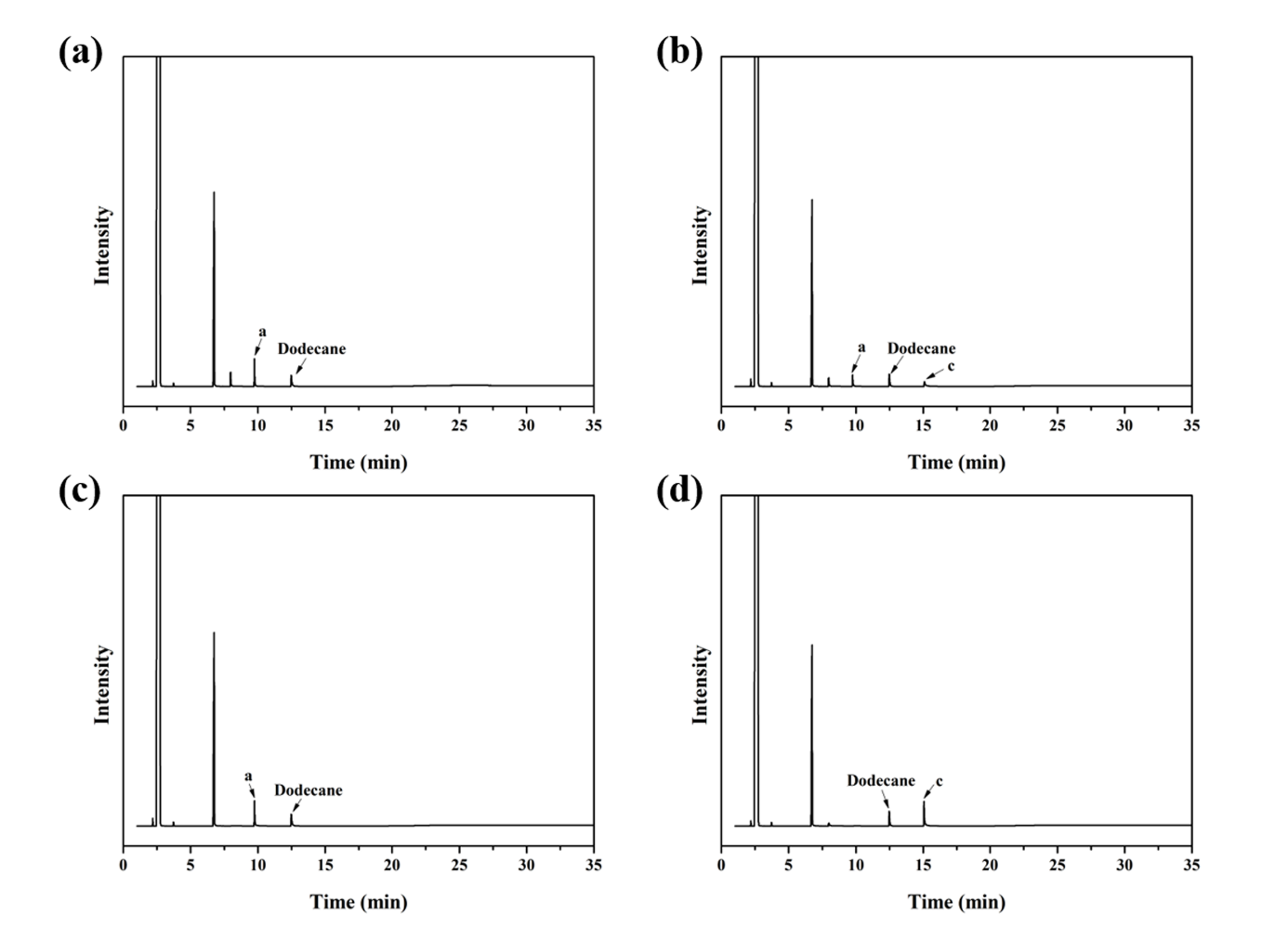


**Figure S13.** GC spectra of the reaction of benzaldehyde to 2-benzylidenemalononitrile before (a, 0 min) and after (b, 1.5 h) catalysis by TpPa powder. GC spectra of the reaction of benzaldehyde to 2-benzylidenemalononitrile before (c, 0 min) and after (d, 1.5 h) catalysis by TpPa sponge-3.

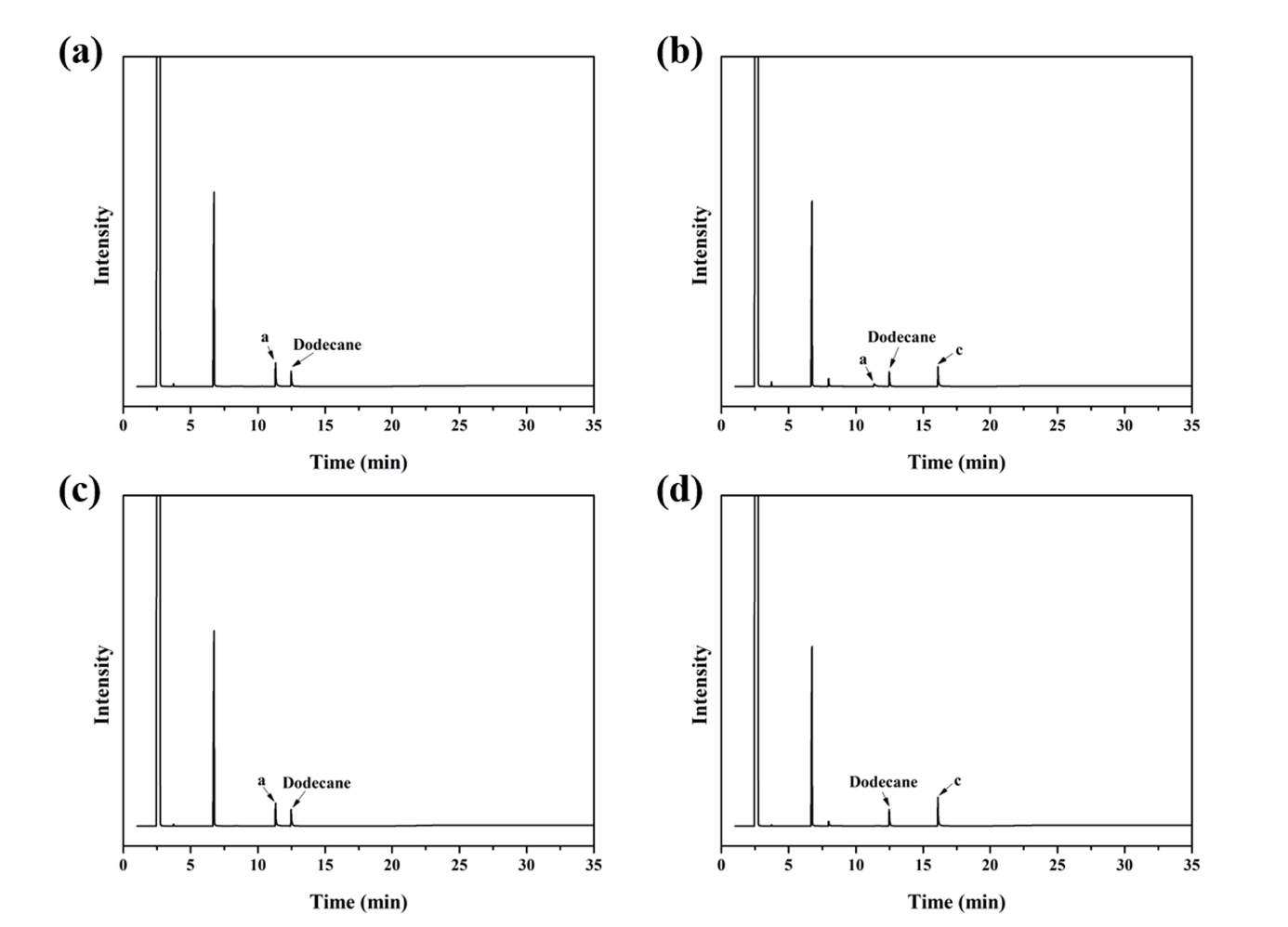


**Figure S14.** GC spectra of the reaction of 4-methylbenzaldehyde to 2-(4-methylbenzylidene) malononitrile before (a, 0 min) and after (b, 0.5 h) catalysis by TpPa powder. GC spectra of the reaction of 4-methylbenzaldehyde to 2-(4-methylbenzylidene) malononitrile before (c, 0 min) and after (d, 0.5 h) catalysis by TpPa sponge-3.

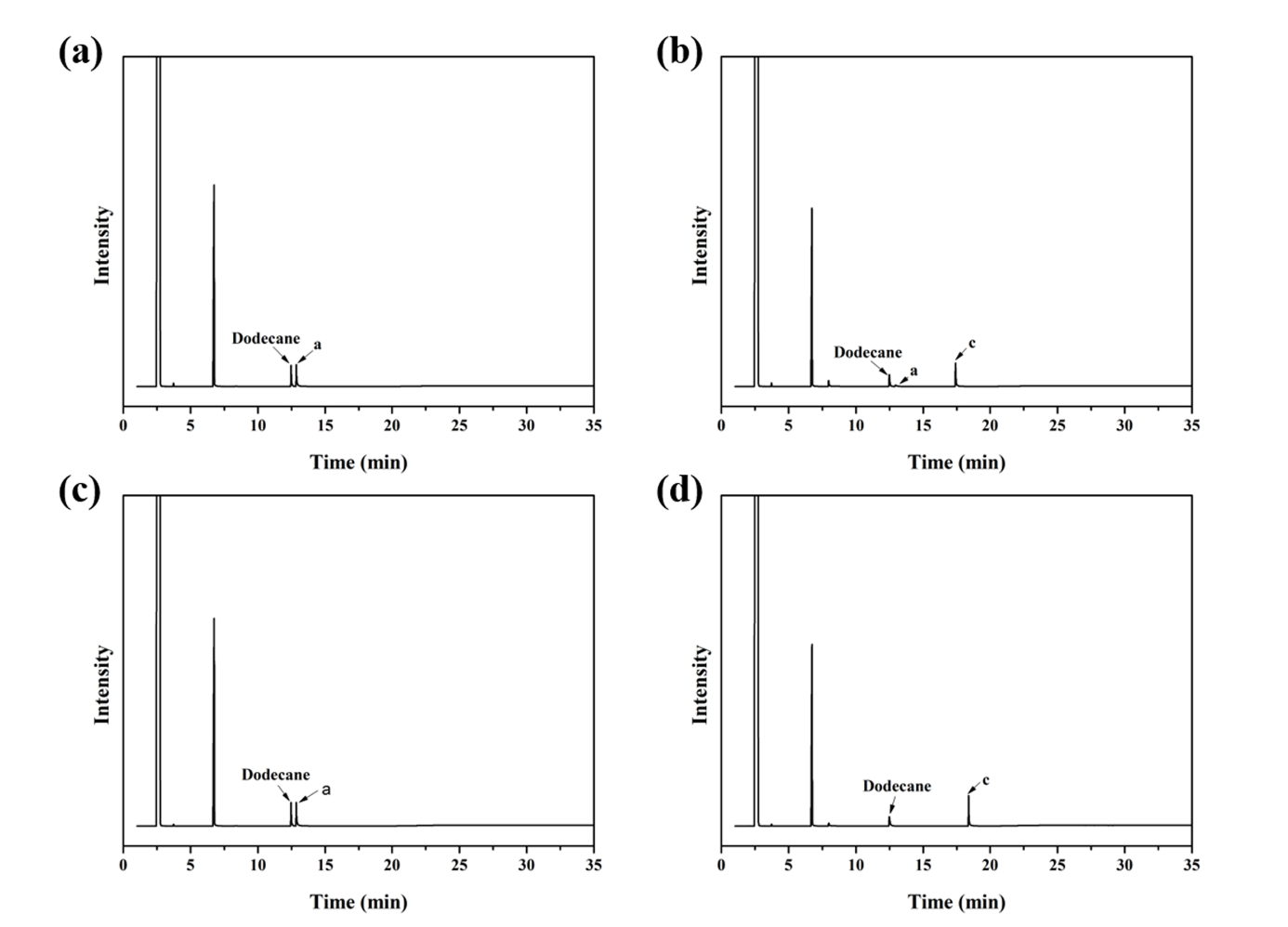


**Figure S15.** GC spectra of the reaction of 4-bromobenzaldehyde to 2-(4-bromobenzylidene) malononitrile before (a, 0 min) and after (b, 0.5 h) catalysis by TpPa powder. GC spectra of the reaction of 4-bromobenzaldehyde to 2-(4-bromobenzylidene) malononitrile before (c, 0 min) and after (d, 0.5 h) catalysis by TpPa sponge-3.

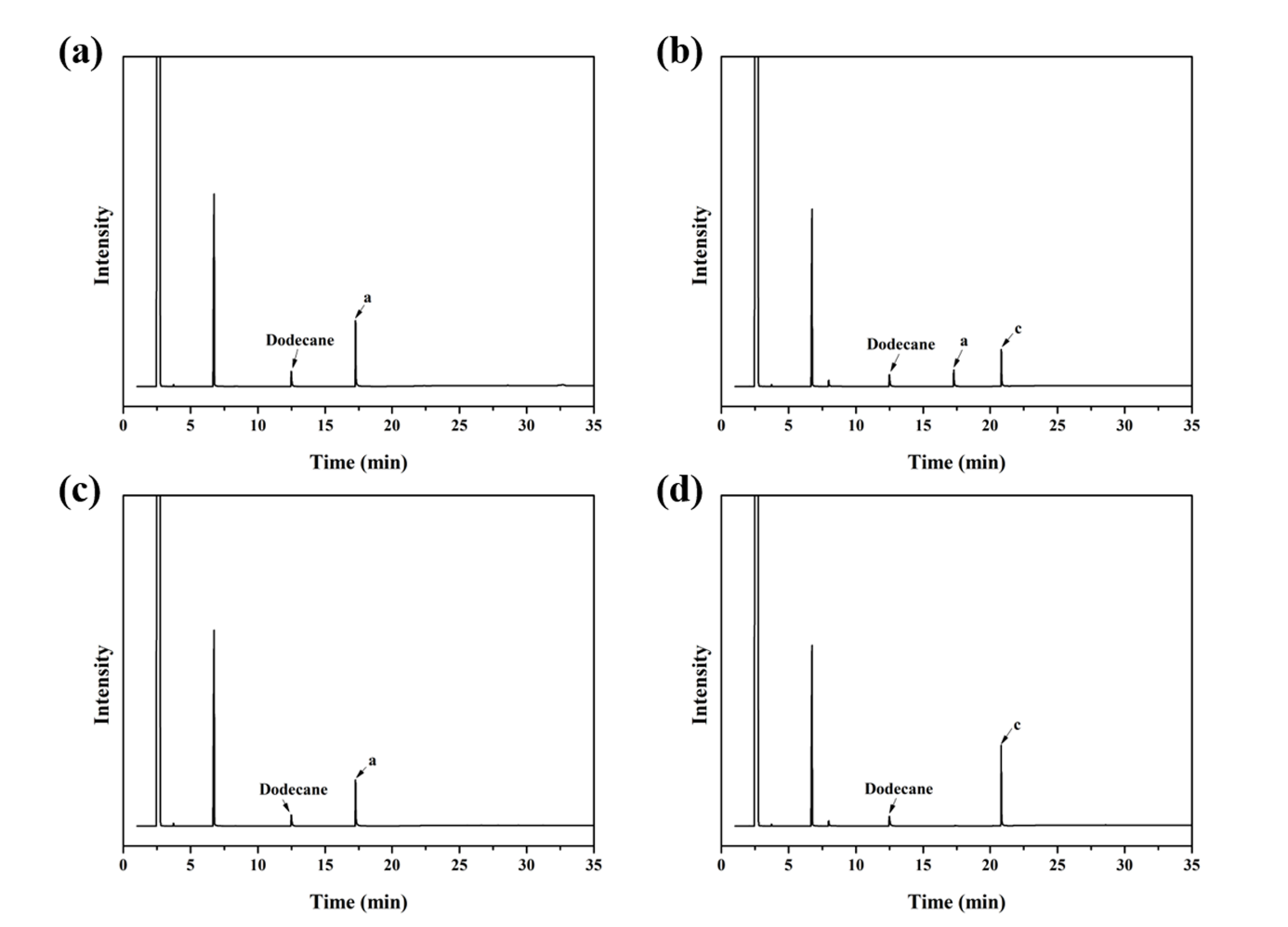


**Figure S16.** GC spectra of the reaction of [1,1'-biphenyl]-4-carbaldehyde to 2-([1,1'-biphenyl]-4-ylmethylene) malononitrile before (a, 0 min) and after (b, 1.5 h) catalysis by TpPa powder. GC spectra of the reaction of [1,1'-biphenyl]-4-carbaldehyde to 2-([1,1'-biphenyl]-4-ylmethylene) malononitrile before (c, 0 min) and after (d, 1.5 h) catalysis by TpPa sponge-3.

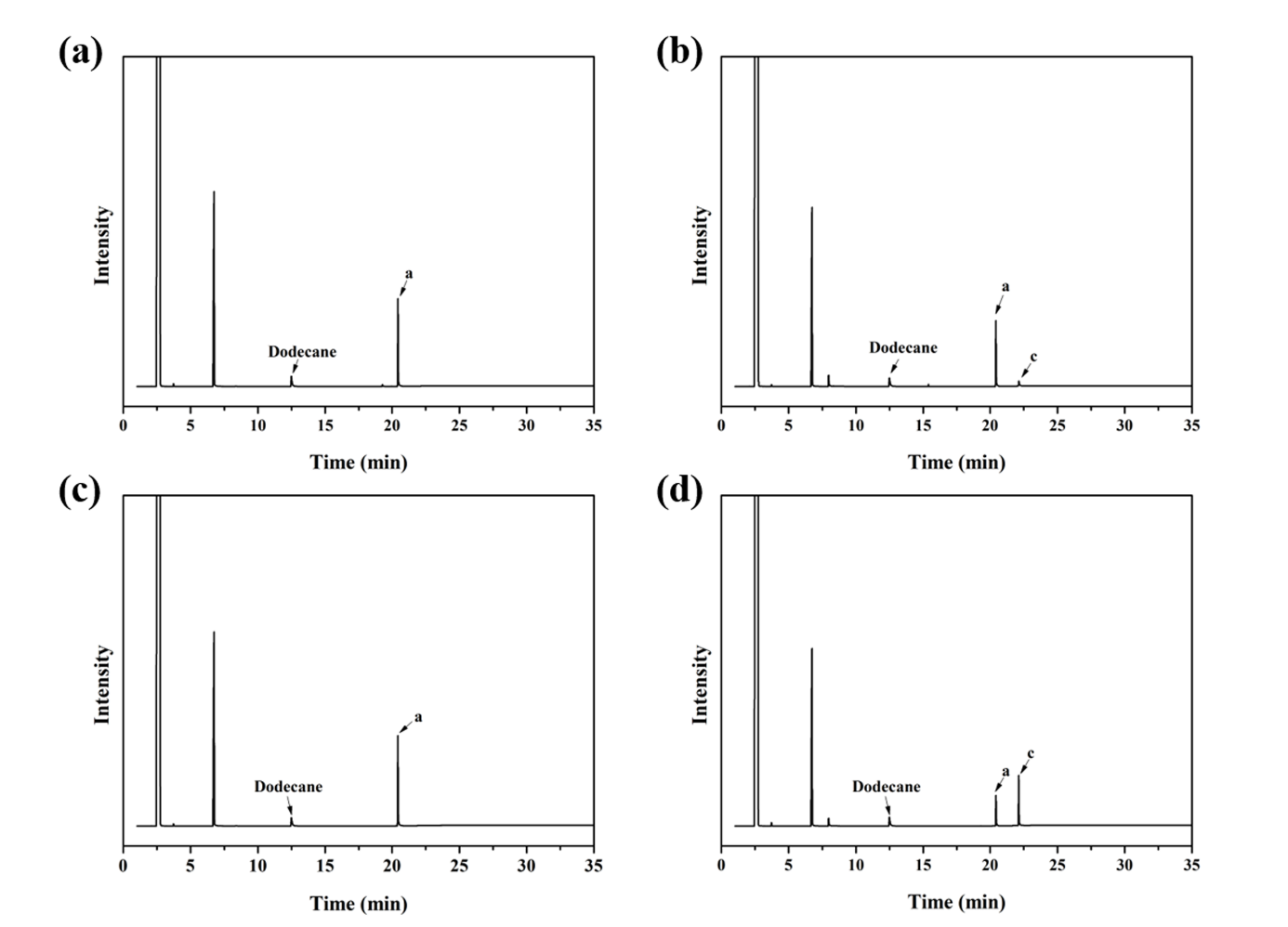


**Figure S17.** GC spectra of the reaction of anthracene-9-carbaldehyde to 2-(anthracen-9-ylmethylene) malononitrile before (a, 0 min) and after (b, 12 h) catalysis by TpPa powder. GC spectra of the reaction of anthracene-9-carbaldehyde to 2-(anthracen-9-ylmethylene) malononitrile before (c, 0 min) and after (d, 12 h) catalysis by TpPa sponge-3.

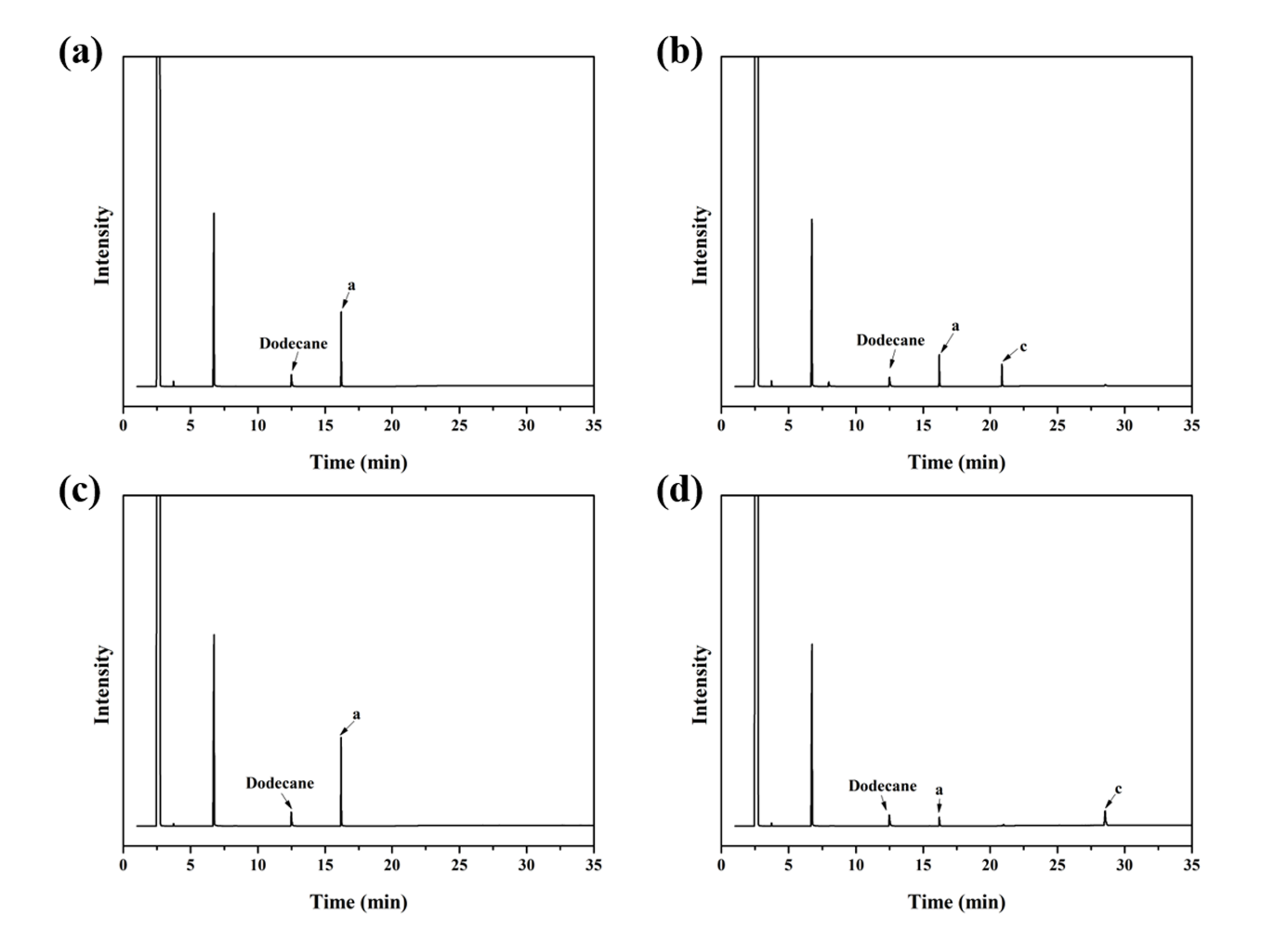


**Figure S18.** GC spectra of the reaction of 3,5-di-tert-butyl-2-hydroxybenzaldehyde to 2-(3,5-di-tert-butyl-2-hydroxybenzylidene) malononitrile before (a, 0 min) and after (b, 12 h) catalysis by TpPa powder. GC spectra of the reaction of 3,5-di-tert-butyl-2-hydroxybenzaldehyde to 2-(3,5-di-tert-butyl-2-hydroxybenzylidene) malononitrile before (c, 0 min) and after (d, 12 h) catalysis by TpPa sponge-3.

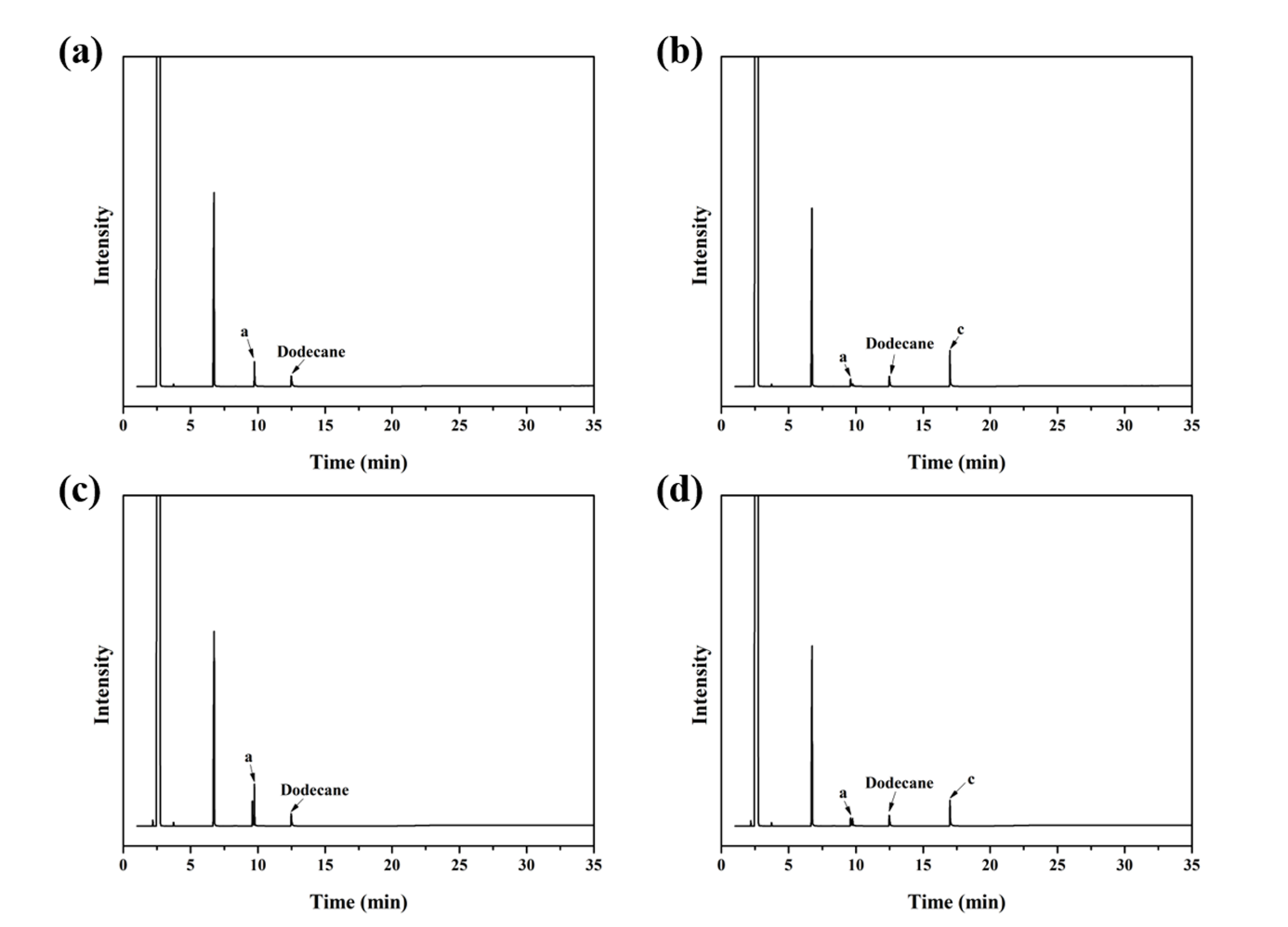


**Figure S19.** GC spectra of the reaction of benzaldehyde to ethyl 2-cyano-3-phenylacrylate before (a, 0 min) and after (b, 6 h) catalysis by TpPa powder. GC spectra of the reaction of benzaldehyde to ethyl 2-cyano-3-phenylacrylate before (c, 0 min) and after (d, 6 h) catalysis by TpPa sponge-3.

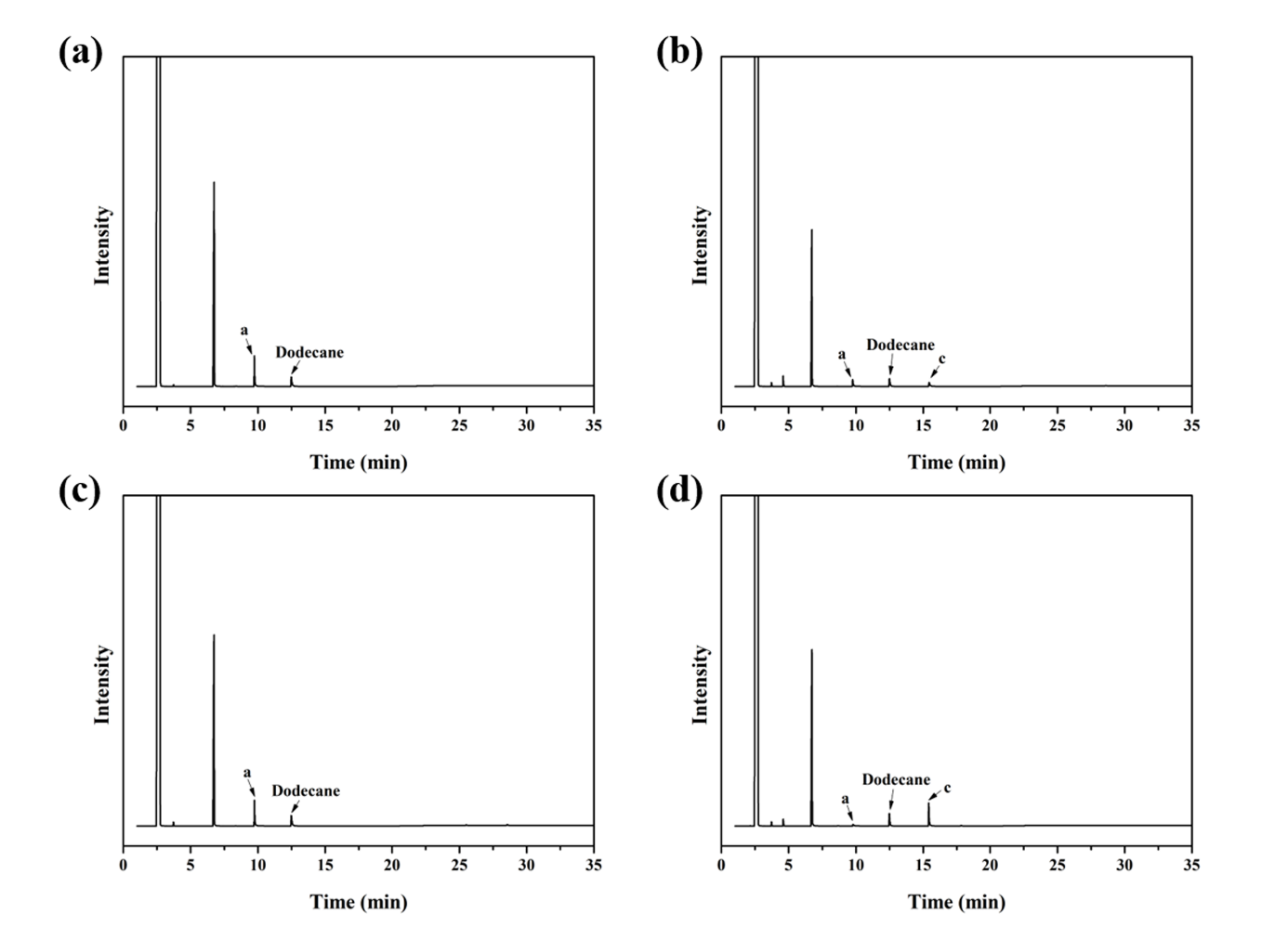


**Figure S20.** GC spectra of the reaction of benzaldehyde to cyano(phenyl)methyl ethyl carbonate before (a, 0 min) and after (b, 6 h) catalysis by TpPa powder. GC spectra of the reaction of benzaldehyde to cyano(phenyl)methyl ethyl carbonate before (c, 0 min) and after (d, 6 h) catalysis by TpPa sponge-3.

**Table S7.** Comparison of the catalytic performance (yield) of TpPa sponge-3 and TpPa powder in various Knoevenagel condensation reactions.

| Substrates  [a] | Substrates  [b] | Products  [c] | Reaction time  [h] | TpPa sponge-3  [%] | TpPa powder  [%] |
| --- | --- | --- | --- | --- | --- |
|  |  |  | 1.5 | 100 | 45.6 |
|  |  |  | 0.5 | 100 | 74.3 |
|  |  |  | 0.5 | 100 | 82.5 |
|  |  |  | 1.5 | 100 | 62 |
|  |  |  | 12 | 63.8 | 31 |
|  |  |  | 12 | 85.1 | 48.3 |
|  |  |  | 6 | 92.7 | 37 |
|  |  |  | 6 | 87.9 | 48.5 |


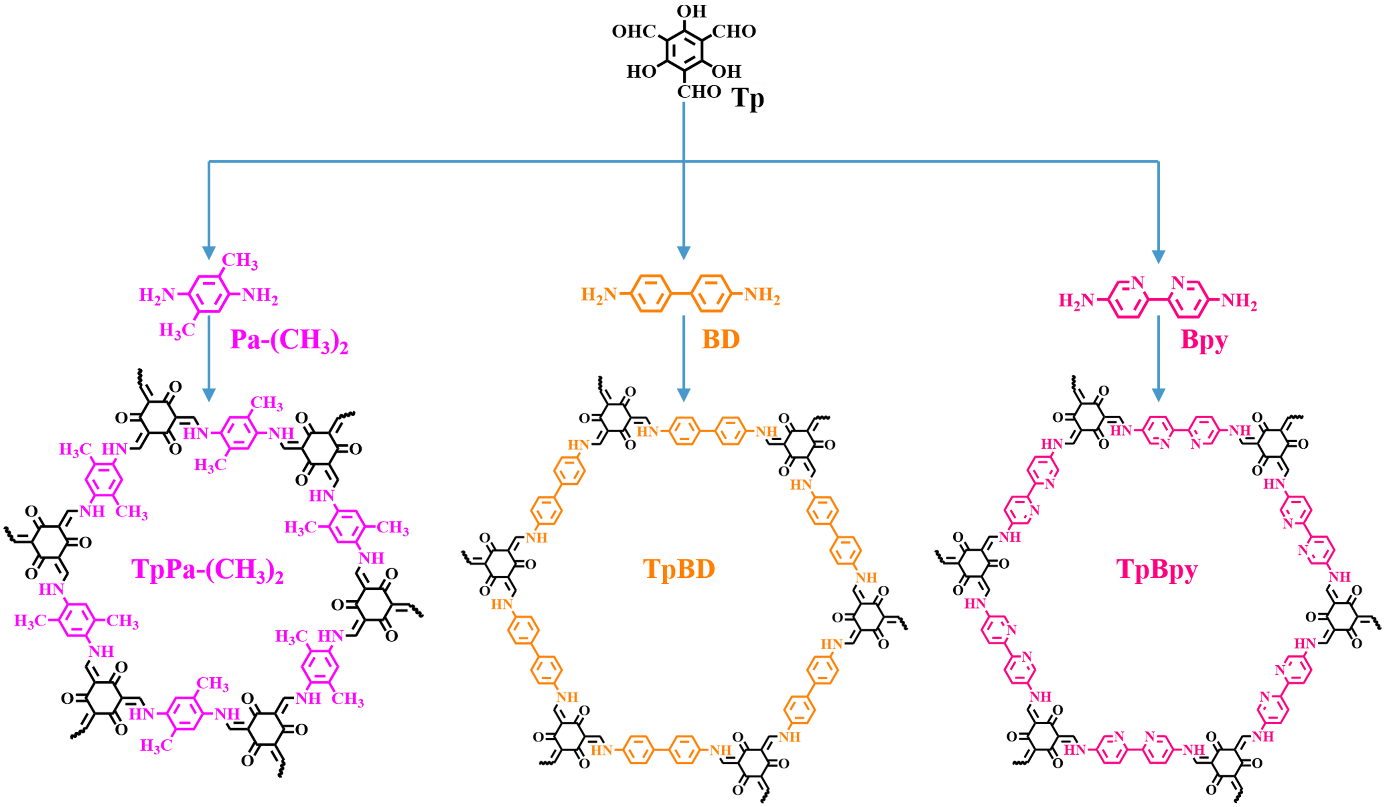


**Figure S21.** Schematic diagram of the synthesis of the other three imine-linked COFs (TpPa-(CH_3_)_2_, TpBD, and TpBpy).


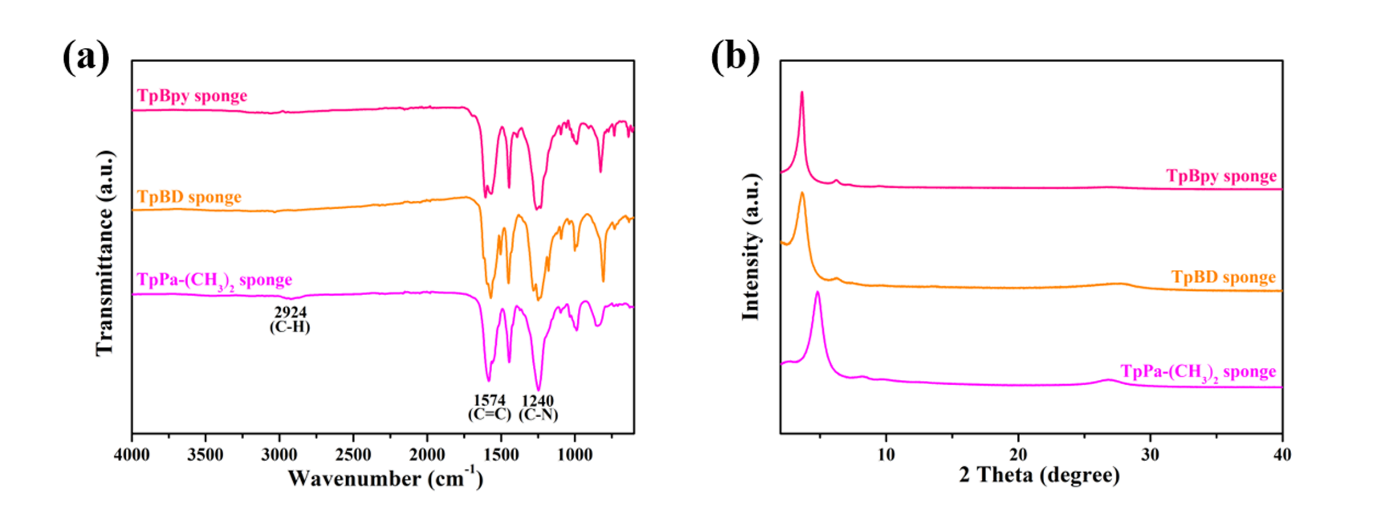


**Figure S22.** FT-IR spectra (a) and XRD patterns (b) of TpPa-(CH_3_)_2_ sponge, TpBD sponge, and TpBpy sponge.


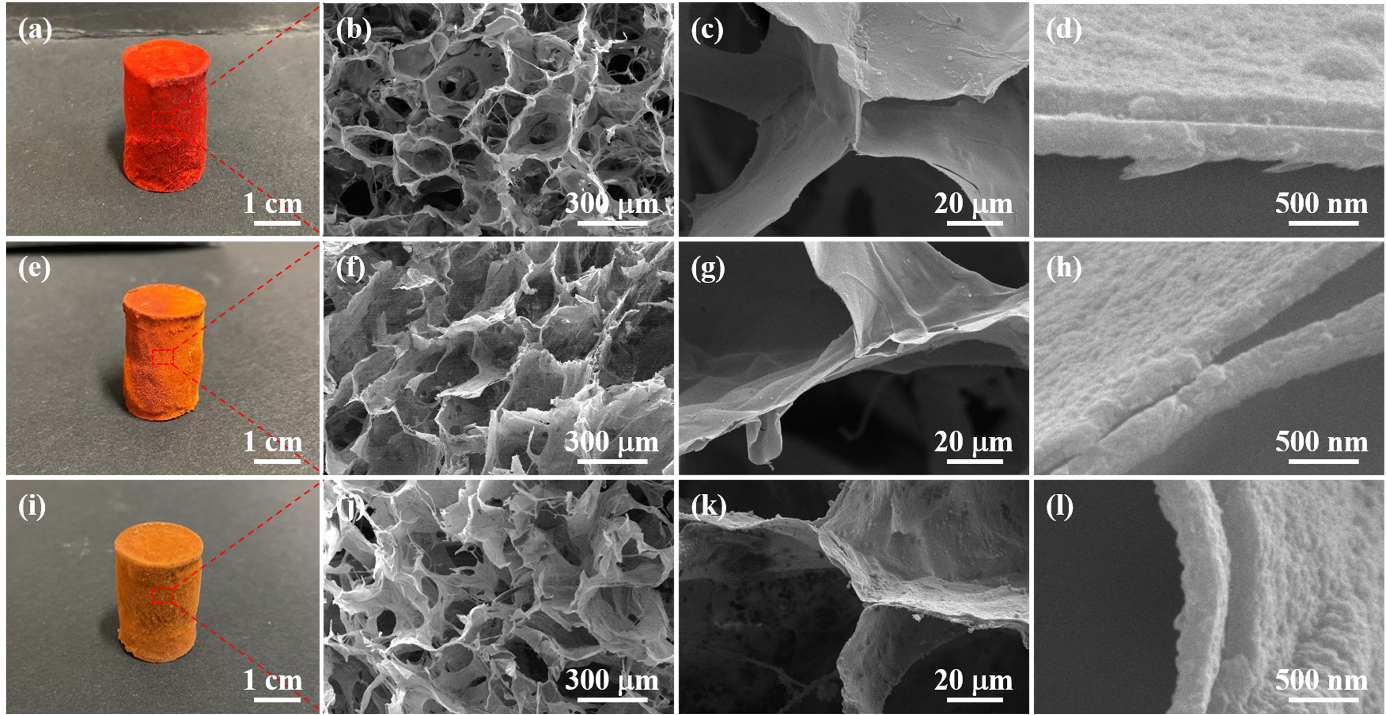


**Figure 23.** Photographs and SEM images of TpPa-(CH_3_)_2_ sponge (a-d), TpBD sponge(e-h), and TpBpy sponge(i-l).

**References**

[1] B. Fan, Y. Si, J. Yu, X. Wang, P. Tang, *Chem. Eng. J.* **2024***,* 155756.

[2] C. X. Li, J. Yang, P. Pachfule, S. Li, M. Y. Ye, J. Schmidt, A. Thomas, *Nat. Commun.* **2020**, *11*, 4712.

[3] C. Li, P. Guggenberger, S. Han, W. Ding, F. Kleitz, *Angew. Chem. Int. Ed.* 2022, **61**, 202206564.

[4] W. Li, W. Jin, H. Jiang, R. Wang, H. Jia, J. Liu, A. Tang, L. Zhu, D. Kong, *Chem. Eng. J.* **2023**, *455*, 140900.

[5] C. Ding, Y. Du, S. Agarwal, *Adv. Funct. Mater.* **2024**, *34*, 2309938.

[6] X. Wang, R. Meng, S. Zhao, Z. Jing, Y. Jin, J. Zhang, X. Pi, Q. Du, L. Chen, Y Li, *Int. J. Biol. Macromol.* **2024**, *260*, 129690.

[7] C. Xiao, Y. Yao, X. Guo, J. Qi, Z. Zhu, Y. Zhou, Y. Yang, J. Li, *Small*, **2024**, 2311881.

[8] F. Li, L. G. Ding, B. J. Yao, N. Huang, J. T. Li, Q. J. Fua, Y. B. Dong, *J. Mater. Chem. A* **2018**, *6*, 11140.

[9] Q. Wang, P. Wang, Y. Wang, Y. Xu, H. Xu, K. Xi, ACS *Appl. Mater. Interfaces* **2024**, *16*, 37052-37062.

[10] J. Martín-Illán, D. Rodríguez-San-Miguel, O. Castillo, G. Beobide, J. Perez-Carvajal, I. Imaz, D. Maspoch, F. Zamora, *Angew. Chem. Int. Ed.* **2021**, *60*, 13969-13977.

[11] X. Li, Z. Jia, J. Zhang, Y. Zou, B. Jiang, Y. Zhang, K. Shu, N. Liu, Y. Li, L. Ma, *Chem. Mater.* **2022**, *34*, 11062-11071.

[12] Q. Wang, L. Gao, P. Wang, Y. Wang, Y. Xu, H. Xu, X. Wang, Z. Meng, K. Xi, *Nanoscale*, **2024**, *16*, 15298-15307.

[13] Y. Wang, H. He, Y. Li, W. Wang, L. Deng, L. Wu, Y. Zhang, J. Huang, P. Zhang, G. Yu, Y. N. Liu, *Matter* **2024**.

[14] F. Hu, Z. Hu, Yu. Liu, K. Tam, R. Liang, Q. Xie, Z. Fan, C. Pan, J. Tang, G. Yu, W. Zhang, *J. Am. Chem. Soc.* **2023**, *145*, 27718-27727.

[15] D. Zhu, Y. Zhu, Q. Yan, M. Barnes, F. Liu, P. Yu, C. Tseng, N. Tjahjono, P. Huang, M. M. Rahman, E. Egap, P. M. Ajayan, R. Verduzco, *Chem. Mater.* **2021**, *33*, 4216-4224.

[16] Q. Ma, L. Zeng, X. Liu, Q. Zhuang, J. Qian, *Microporous Mesoporous Mater.* **2022**, *331*, 111623.

[17] L. G. Ding, B. J. Yao, F. Li, S. C. Shi, N. Huang, H. B. Yin, Q. Guan, Y. B. Dong, *J. Mater. Chem. A* **2019**, *7*, 4689-4698.

[18] Q. Li, Y. Cui, Y. Xiao, Z. Ni, S. Dai, F. Chen, C. Guo, *Talanta* **2024**, *275*, 126088.

[19] Z. Zhang, X. Shi, X. Wang, Z. Zhang, Y. Wang, *Sep. Purif. Technol.* **2023**, *309*, 123108.

[20] Q Li, S Zhu, F Wu, F Chen, C Guo, *Microchimica Acta* **2023**, *190*, 369.

[21] P. Zhang, Z. Wang, S. Wang, J. Wang, J. Liu, T. Wang, Y. Chen, P. Cheng, Z. Zhang, *Angew. Chem. Int. Ed.* **2022**, *61*, e202213247.

[22] Y. Si, J. Yu, X. Tang, J. Ge, B. Ding, *Nat. Commun.* **2014**, *5*, 5802.

[23] B. Yao, J. Chen, L. Huang, Q. Zhou, G. Shi, *Adv. Mater.* **2015**, *28*, 1623.

[24] S. Long, Y. Feng, F. He, S. He, H. Hong, X. Yang, L. Zheng, J. Liu, L. Gan, M. Long, *Carbon* **2020**, *158*, 137.

[25] L. Dou, X. Zhang, H. Shan, X. Cheng, Y. Si, J. Yu, B. Ding, *Adv. Funct. Mater.* **2020**, *30*, 2005928.

[26] H. Zhuo, Y. Hu, X. Tong, Z. Chen, L. Zhong, H. Lai, L. Liu, S. Jing, Q. Liu, C. Liu, X. Peng, R. Sun, *Adv. Mater.* **2018**, *30*, 1706705.

[27] X. Wang, L. L. Lu, Z. L. Yu, X. W. Xu, Y. R. Zheng, S. H. Yu, *Angew. Chem. Int. Ed.* **2015**, *54*, 2397.

[28] L. Su, H. Wang, M. Niu, X. Fan, M. Ma, Z. Shi, S. Guo, *ACS Nano* **2018**, *12*, 3103.

[29] B. Qin, Z. L. Yu, J. Huang, Y. F. Meng, R. Chen, Z. Chen, S. H. Yu, *Angew. Chem. Int. Ed.* **2023**, *62*, e202214809.

[30] J. R. Guo, S. B. Fu, Y. P. Deng, X. Xu, S. J. Laima, D. Z. Liu, P. Y. Zhang, J. Zhou, H. Zhao, H. X. Yu, S. X. Dang, J. N. Zhang, Y. D. Zhao, H. Li, X. F. Duan, *Nature* **2022**, *606*, 909.

[31] X. Chang, F. Wu, X. Cheng, H. Zhang, L. He, W. Li, X. Yin, J. Yu, Y. T. Liu, B. Ding, *Adv. Mater.* **2023**, *36*, 2308519.

[32] H. Guo, Q. Fei, M. Lian, T. Zhu, W. Fan, Y. Li, L. Sun, F. De Jong, K. Chu, W. Zong, C. Zhang, T. Liu, *Adv. Mater.* **2023**, *35*, 2301418.

[33] J. Zhang, B. Li, L. Li, A. Wang, *J. Mater. Chem. A* **2016**, *4*, 2069.

[34] L. Li, B. Li, H. Sun, J. Zhang, *J. Mater. Chem. A* **2017**, *5*, 14858-14864.

[35] Y. Si, X. Wang, C. Yan, L. Yang, J. Yu, B. Ding, *Adv. Mater.* **2016**, *28*, 9512.

[36] H. Lai, H. Zhuo, Y. Hu, G. Shi, Z. Chen, L. Zhong, M. Zhang, *ACS Sustainable Chem. Eng.* **2021**, *9*, 9761.

[37] Y. Du, C. Ding, S. Agarwal, *Adv. Energy Sustain. Res.* **2024**, *5*, 2300218.

[38] Y. Hu, H. Zhuo, Z. Chen, K. Wu, Q. Luo, Q. Liu, S. Jing, C. Liu, L. Zhong, R. Sun, X. Peng, *ACS Appl. Mater. Interfaces* **2018**, *10*, 40641.

[39] H. Y. Mi, X. Jing, A. L. Politowicz, E. Chen, H. X. Huang, L. S. Turng, *Carbon* **2018**, *132*, 199.

[40] Z. Chen, H. Zhuo, Y. Hu, H. Lai, L. Liu, L. Zhong, X. Peng, *Adv. Funct. Mater.* **2020**, *30*, 1910292.

[41] C. Wang, X. Chen, B. Wang, M. Huang, B. Wang, Y. Jiang, R. S. Ruoff, *ACS Nano* **2018**, *12*, 5816.

[42] P. Zhang, Z. Wang, Y. Yang, S. Wang, T. Wang, J. Liu, P. Cheng, Y. Chen, Z. Zhang, *Sci. China Chem.* **2022**, *65*, 1173-1184.

[43] N. Han, Z. Zhang, H. Gao, Y. Qian, L. Tan, C. Yang, H. Zhang, Z. Cui, W. Li, X. Zhang, *ACS Appl. Mater. Interfaces* **2020**, *12*, 2926.

[44] Q. Sun, B. Aguila, J. A. Perman, T. Butts, F.-S. Xiao, S. Ma, *Chem* **2018**, *4*, 1726.

[45] J. Li, Y. Yang, W. Ma, G. Li, Z. Lin, *J. Hazard. Mater.* **2021**, *411*, 125190.

[46] Y. Liu, Q. Lyu, Z. Wang, Y. Sun, C. Li, S. Sun, L. C. Lin, S. Hu, *J. Mater. Sci.* **2021**, *56*: 13031-13042.

[47] H. Bi, X. Xie, K. Yin, Y. Zhou, S. Wan, L. He, F. Xu, F. Banhart, L. Sun, R. S. Ruoff, *Adv. Funct. Mater.* **2012**, *22*, 4421.

[48] J. Gu, H. Fan, C. Li, J. Caro, H. Meng, *Angew. Chem. Int. Ed.* **2019**, *131*, 5351-5355.

[49] X. Gui, J. Wei, K. Wang, A. Cao, H. Zhu, Y. Jia, Q. Shu, D. Wu, *Adv. Mater.* **2010**, *22*, 617.

[50] X. Dong, J. Chen, Y. Ma, J. Wang, M. B. Chan-Park, X. Liu, L. Wang, W. Huang, P. Chen, *Chem. Commun.* **2012**, *48*, 10660.
